# Development of a Unified Specimen for Adhesive Characterisation—Part 1: Numerical Study on the Mode I (mDCB) and II (ELS) Fracture Components

**DOI:** 10.3390/ma16082951

**Published:** 2023-04-07

**Authors:** Daniel S. Correia, Inês D. Costa, Beatriz D. Simões, Eduardo A. S. Marques, Ricardo J. C. Carbas, Lucas F. M. da Silva

**Affiliations:** 1 Institute of Science and Innovation in Mechanical and Industrial Engineering (INEGI), University of Porto, Rua Dr. Roberto Frias 400, 4200-465 Porto, Portugal; 2 Department of Mechanical Engineering, Faculty of Engineering (FEUP), University of Porto, Rua Dr. Roberto Frias 400, 4200-465 Porto, Portugal

**Keywords:** structural adhesives, adhesive characterisation, fracture toughness, fracture process zone, unified specimen

## Abstract

Adhesives are increasingly being employed in industrial applications as a replacement for traditional mechanical joining methods, since they enable improvements in the strength-to-weight ratio and lower the cost of the overall structures. This has led to a need for adhesive mechanical characterisation techniques that can provide the data needed to build advanced numerical models, allowing structural designers to expedite the adhesive selection process and grant precise optimisation of bonded connection performance. However, mechanically mapping the behaviour of an adhesive involves numerous different standards resulting in a complex network of various specimens, testing procedures and data reduction methods that concern techniques which are exceedingly complex, time-consuming, and expensive. As such, and to address this problem, a novel fully integrated experimental characterisation tool is being developed to significantly reduce all the issues associated with adhesive characterisation. In this work, a numerical optimisation of the unified specimen’s fracture toughness components, comprising the combined mode I (modified double cantilever beam) and II (end-loaded split) test, was performed. This was achieved by computing the desired behaviour as a function of the apparatus’ and specimens’ geometries, through several dimensional parameters, and by testing different adhesives, widening the range of applications of this tool. In the end, a custom data reduction scheme was deduced and set of design guidelines was defined.

## 1. Introduction

In many industries, including those operating in the automotive, aerospace, and aviation sectors, adhesive bonding technology has increasingly become a viable substitute for traditional mechanical joining methods. Adhesively bonded joints offer several significant advantages over other common joining techniques such as bolting and riveting, a more uniform stress distribution along the bonded area which avoids stress concentrations and improves load transmission and fatigue resistance. Other important benefits include this technology’s capacity to easily bond dissimilar materials together and the increased flexibility it provides to the joint design [1]. As a result of these advantages, structural weight and cost reductions can be obtained with the use of well-designed bonded joints.

The growth in the use of adhesives, especially those of the structural nature, has led to a need for fast and effective adhesive mechanical characterisation techniques that can provide the data needed to help structural designers in the adhesive selection process and grant precise optimisation of bonded connection performance. Characterising the mechanical properties and knowing the limitations of each adhesive provides crucial insights for ensuring quality control, enhancing bond performance and facilitating the usually challenging adhesive selection process [1]. Currently, multiple different tests must be performed to obtain a set of the most relevant mechanical properties for joint design, such as tensile tests and shear tests, for strength and stiffness, and fracture tests in both mode I and mode II, to determine the toughness of the adhesive.

Tensile properties are commonly determined through bulk tensile tests and butt joints [2]. The ASTM D 897 and ASTM D 2095 standards describe directives related to the practical aspects and methodology of preparing and testing to perform this type of test. The first one uses short circular specimens made of metal or wood, and the second is more general and includes round and square geometries [2].

For shear properties, the thick adherend shear test (TAST) appears as a common choice, since it relies on an easily produced specimen and a simple testing setup [2]. It also emerges as a valid substitute for the conventional single lap shear (SLS) joint, which although simple to execute is beset with complex stress states and, therefore, is not suitable for characterisation of the strength properties of adhesives. TAST tests can be performed using two different standards, ASTM D3983 and ISO 11003-2, where the main difference is in the size of the specimen. However, difficulties concerning the stress concentrations generated due to edge effects have been reported, directly influencing the acquisition of reliable data. In order to acquire accurate experimental results, a modified TAST fixture was created by Cognard et al. [3], although it is still not standardised. It provides a stiffer apparatus combined with a more compact specimen, resulting in a more uniform adhesive stress condition in the joint and severe restriction of edge effects [2].

**Figure 1:**
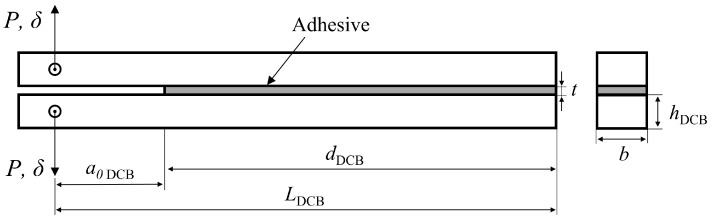
Schematic representation of the DCB specimen and its associated testing procedure.

The most popular test specimens for determining the critical energy release rate in mode I (the tensile opening mode), i.e., $G_{IC}$, are the double cantilever beam (DCB), Figure 1, and the tapered double cantilever beam (TDCB) adhesive joint test specimens. In contrast to the more complex and expensive specimens used in TDCB testing, the DCB test uses simpler, less expensive specimens. Although the TDCB has the advantage of showing a linear change in compliance with crack length [4], this only makes them effective in situations where it is not practical or desirable to measure crack lengths while carrying out the test. However, even though Blackman et al. [5] showed that the values of $G_{IC}$ obtained through these tests were independent of the test’s geometry, it is often better to use simpler specimens, and, as a result, DCB tests are commonly preferred. Furthermore, multiple crack independent data reduction schemes have been developed for DCB tests, which further simplifies the use of this configuration [6], by removing the need to directly monitor crack propagation. Both these tests have been studied since the 1960s and the work of different authors has led to the publication of an ASTM D3433 standard in 1973. Being revised by Blackman et al. [5] through an inter-laboratory round-robin test programme, this resulted in the publication of BS 7991 in 2001. More recently, due to the increased popularity of fiber-reinforced polymer matrix composites, a new international standard has been published, named ISO 25217 [2].

**Figure 2:**
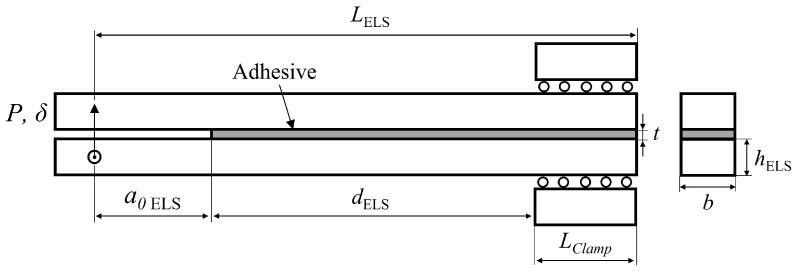
Schematic representation of the ELS specimen and its associated testing procedure.

On the other hand, only the testing of composites is currently standardised for pure mode II (the in-plane shearing mode) fracture toughness ($G_{IIC}$) characterisation by means of the end-notched flexure (ENF) test, although this process has been successfully modified for adhesive joint testing. The end-loaded split (ELS) is also proposed and widely used for the determination of mode II fracture toughness [7,8], Figure 2. The most common mode II tests do, however, still have certain limitations.

Under shear loads, especially for ductile adhesives, bonded joints have been reported to have a large fracture process zone (FPZ) compared to mode I or mixed-mode fracture tests and compared to other specimen dimensions, owing to the adhesive layer’s plasticity. The large FPZ involved, along with the lack of crack opening, makes visual evaluation of the crack length difficult. To address this, approaches based on J-integral [9] or an effective crack length ($a_{eq}$) that are non-crack dependent have been developed [6,10,11,12,13,14,15]. Furthermore, the commonly used end-notched flexure (ENF) test presents instability problems associated with the crack propagating in the direction of the highest flexural moment, so other tests have been developed to avoid this effect, including the tapered ENF (TENF) test, the stabilised ENF (SENF) test, the over-notched flexure (ONF), the four-point ENF (4ENF), or the inverse ELS (I-ELS) [16] test, among others, to achieve stable propagation. However, other issues arise from these tests, such as challenges with the production of the specimens (TENF), the complexity of the test setup (SENF), or friction-related issues (4ENF and ONF). The end-loaded split tests, in addition to having simpler manufactured specimens, based on DCB specimens, have shown encouraging results in recent studies, such as the advantage of having stable crack propagation under displacement control when the ratio between the initial crack and the span length is higher than 0.55, in composite joints [12,17,18,19,20].

Recent research has shown that certain criteria must be met in order to obtain stable crack propagation during an ELS test. First, it is necessary ensure formation of the entire FPZ by reaching steady-state crack propagation and, as a result, the R-curve plateau; second, to ensure the test’s stability under displacement control; third, to avoid significant deflections; and fourth, to avoid adherend failure during testing. The span length ($L_{\mathrm{ELS}}$), initial crack length (${a_{0}}_{\mathrm{ELS}}$), and, if necessary, height ($h_{\mathrm{ELS}}$) of the ELS specimen geometry must be changed in order to satisfy these requirements [21,22].

Contrarily to the strength tests, fracture tests rely heavily on procedures of data reduction to extract from the *P*-δ curves the material’s energy release rate, $G_{C}$. There are two main types of formulations that can be devised, based either on Linear Elastic Fracture Mechanics (LEFM), i.e., the Compliance-based Beam Method (CBBM), or Non-linear Fracture Mechanics (NLFM), i.e., the J-integral.

Some of the classical LEFM data reduction schemes used to obtain fracture energies [6,23], based on the Irwin–Kies equation, are the Compliance Calibration Method (CCM), the Direct Beam Theory (DBT), and the Corrected Beam Theory (CBT), with this last one having the correction factor Δ, proposed in 1992 by Wang and Williams [24,25], to account for the crack tip rotation and deflection.All of these approaches require measuring the crack length, which can be difficult, especially in Mode II and when the crack propagates suddenly. When extensive damage occurs, such as microcracking or fiber bridging, this also becomes a problem [26]. More recently, the CBBM [6,11] approach, based on an equivalent crack length concept, was used to characterise the fracture of bonded joints by developing a data reduction scheme method that took into account the Timoshenko beam theory and the specimen’s compliance. This proposed methodology eliminates the need to measure the crack length during propagation and accounts for FPZ effects, which are especially important in ductile adhesives and mode II loading. Furthermore, this data reduction method can be applied to Mode I [6], Mode II [11] and Mixed Mode [27,28].

As for the NLFM approaches, the J-integral, whose formulation was developed for elastic materials in the late 60’s by Cherepanov [29] and Rice [30], is the most well known and is based on the application of a contour integral. Rice also demonstrated that, as long as the deformation theory of plasticity is used, the J-integral is independent of the contour for any elastic or elasto-plastic material [31]. As a result, several researchers [32,33,34] were able to use this approach when studying ductile materials. Therefore, the J-integral is only valid in materials with a non-linear stress–strain relation if there is no unloading in any point of the material, implying that the stress–strain relation is non-linear elastic [35]. Furthermore, the J-integral can be used in all loading modes, including Mode I [34,35,36], Mode II [33,37,38], and Mixed Mode [39,40,41].

Sun and Blackman [36] employed several methods to obtain the fracture toughness of three distinct adhesives and assessed their applicability in each situation in a Mode I study in which $G_{C}$, $J_{C}$, and the cohesive laws were all simultaneously determined. The authors discovered that for the brittle adhesive and toughened epoxy, all of the $G_{C}$ values obtained using the LEFM method agreed with the $J_{C}$ values, proving their validity. The $G_{C}$ values for a ductile polyurethane adhesive, on the other hand, were 15% higher than the $J_{C}$, values, indicating that the same methods are not valid. Furthermore, the same authors used Digital Image Correlation (DIC) to automate the measurement of crack length and fracture toughness in Mode I with structural adhesives. They discovered that for all three adhesives, the CBBM and the CBT with a crack length extended to the compression zone produced G values very similar to J. More specifically, they found nearly identical values for all CBT cases and one adhesive in CBBM, while the remaining two showed a 6% increase in comparison to the J values.

Although there are several specimens developed to characterise the mechanical behaviour of adhesives to be studied, so far there are no tests that combine more than one loading condition simultaneously. Ultimately, to fully mechanically characterise an adhesive the following is necessary: four specimens, four testing procedures and four data reductions schemes. In addition to having to perform the four tests separately with their specific apparatuses, it is also mandatory to have the knowledge on how to properly manufacture, test and treat the obtained data, making adhesive characterisation a complex, time-consuming and costly procedure for non-specialised personnel.As such, from an industrial point of view, companies could increase their competitiveness by not having to hire third parties for this purpose by performing all tests in house.

With this in mind a novel experimental tool [42], that sequentially loads a unified specimen in the four previously mentioned loading scenarios in a single test, is being developed at INEGI (Porto, Portugal). This is performed by combining a butt joint for tensile strength, the modified TAST concept for shear strength, the DCB for mode I fracture toughness, and the ELS—for mode II fracture toughness. Other than the novel specimen and apparatus, the goal of this project is to simplify the adhesive characterisation procedure as much as possible in all aspects, manufacturing, testing and data reduction.

Thus, the present work focuses on part of the bigger picture, the development of an intermediate concept, in which both fracture tests required to characterise the adhesive in mode I and mode II can be performed with a single test.

**Figure 3:**
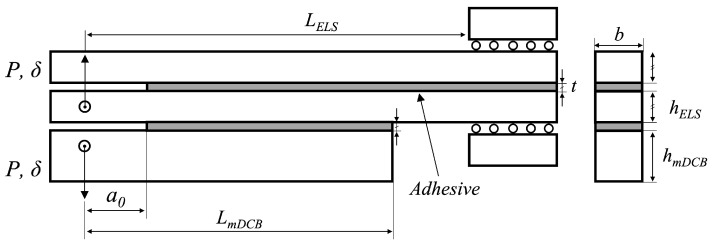
Schematic representation of the unified mode I and II fracture specimen and its associated testing procedure.

In this manuscript, the fracture component of the unified specimen was numerically tested, combining the DCB test for mode I, and the ELS test for mode II, resulting in the specimen presented in Figure 3. Due to the combined character of the novel concept, a new mode I test is defined, as a modified DCB (mDCB) test, where the usually solid double cantilever beams are replaced by a solid beam on the bottom and the full-fledged ELS specimen on top, as seen in Figure 3.

As a result of the significant discrepancy between the cross-head displacements required to have mode I or mode II propagation, and being tested with a specially designed apparatus, mode I and mode II cracks propagate independently and in that order. This enables each test to be analysed separately, considering the whole specimen for mode I, and only the ELS for mode II, since at this stage the mDCB’s bottom bar has completely debonded from the composed specimen.

This work was performed using two structural epoxy adhesives of distinct nature, one being brittle and the other tough, to validate the applicability range of the specimen. Additionally, the study was divided into two steps, starting with the ELS test since it influences the mode I component, and then the mDCB test was analysed as a whole. This was done to isolate each fracture mode and understand their isolated particularities. As such, the ELS specimen geometry was firstly analysed by computing behaviour changes as a function of the specimen’s dimensions and assessing the limitations of this test associated to the existence of a clamp tool. Finally, the study concludes with an analysis of the geometry of the novel modified DCB test, focusing mainly on the influence of the stiffness discontinuity present in the top composed beam (ELS specimen) on the mode I characterisation process, comparing it with the standard DCB test, as a benchmark reference.

As an outcome, several guidelines for the design of this unified specimen for combined mode I and mode II fracture characterisation are proposed, and a custom mode I data reduction method for the mDCB specimens was successfully employed.

## 2. Numerical Details

**Figure 4:**
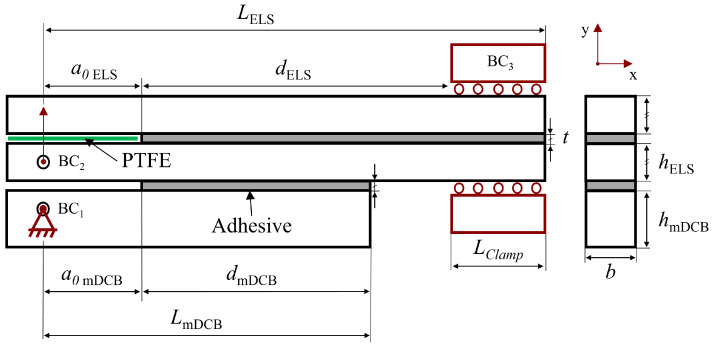
Schematic representation of the mDCB and ELS fracture specimen, with its associated dimensions, testing procedure and respective boundary conditions.

In the scope of this study, a combined test intended to be used for the extraction of mode I and mode II energy release rate, merging a double cantilever beam with an end-loaded split joint—Figure 4—is numerically analysed. Since these configurations are sequentially tested (first the mode I part and then the mode II component) they can be examined separately.

Since the mode I modified double cantilever beam test is dependent on the end-loaded split specimen geometry, as seen in Figure 4, the mode II ELS test will be analysed first as an isolated specimen; followed by the combined specimen solely considering the mode I mDCB test, whose pure mode I performance will be compared against the standard DCB test.

Having already established the geometries in use, ELS in Figure 2, and mDCB in Figure 4 versus DCB in Figure 1, in this section, the numerical model used for both fracture mode tests is described, focusing on common aspects and then advancing to specific considerations relative to the study of the ELS test and the mDCB test.

### 2.1. Finite Element Model

The selection of the numerical model configuration, element types used in the mesh and other numerical parameters will strongly influence the result’s accuracy, requiring a careful definition of the model parameters to ensure accuracy.

Due to the non-linear character of the simulation at hand and to simplify the study, a 2D planar analysis, considering auto adjustable increment with a maximum increment of 0.5% of the applied displacements, was performed to obtain smooth crack propagation. The non-linearities associated with adhesive crack propagation were also taken into consideration by implementing cohesive element following a triangular cohesive zone model (CZM).

#### 2.1.1. Partitions

**Figure 5:**
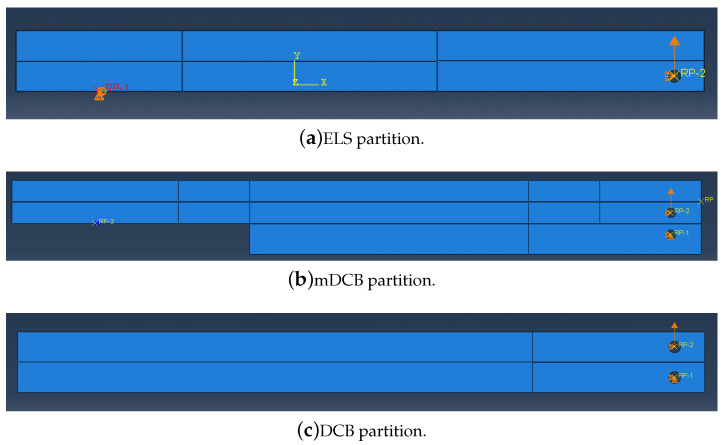
Representative partitions of the specimens used in this numerical study. The boundary conditions are also present in these figures.

In complex models, the definition of small partitions of the model is commonly carried out by dividing it into smaller areas, to whom each element type and size could be attributed, ensuring that a regular and homogeneous mesh could be created for the model. Such representative areas can be seen in Figure 5, for the ELS (5a), mDCB (5b) and DCB (5c) specimens.

#### 2.1.2. Mesh

Concerning the mesh elements, since this was a 2D simulation, the elements considered for the steel substrates were four-node bilinear plane strain elements (CPE4R) with reduced integration.

Additionally, taking into account the fact that cohesive zone models were used to simulate the adhesive, four-node two-dimensional cohesive elements (COH2D4) were used.

All mesh partitions were set as structured except for the loading hole partition, which remained a freely meshed region. The mesh used to simulate this specimen was generally composed of quadrilateral elements ranging from 0.5 mm to 1 mm side. Biased meshing was implemented in the direction of crack propagation, changing from 0.5 mm at the crack tip to 1 mm by the end of the respective partitions. All these parameters were tailored in order to avoid convergence issues.

#### 2.1.3. Cohesive Element Formulation

A cohesive material behaviour based on the model proposed by Alfano and Crisfield [43] was considered to simulate damage initiation and propagation. The constitutive relationship is established between the vector of stresses (σ) and relative displacements (δ) in each moment, defining the traction–separation law.

Cohesive elements are often defined with null thickness. However, this model is adapted for a finite thickness to ensure that the elements capture the elastic behaviour, prior to damage initiation. An energy-based damage evolution was associated to the cohesive material, as it defines damage in terms of the energy required to reach failure after the initiation of damage, i.e., fracture energy. This method is commonly found in the literature [44], as this energy value can be directly and experimentally determined.

**Figure 6:**
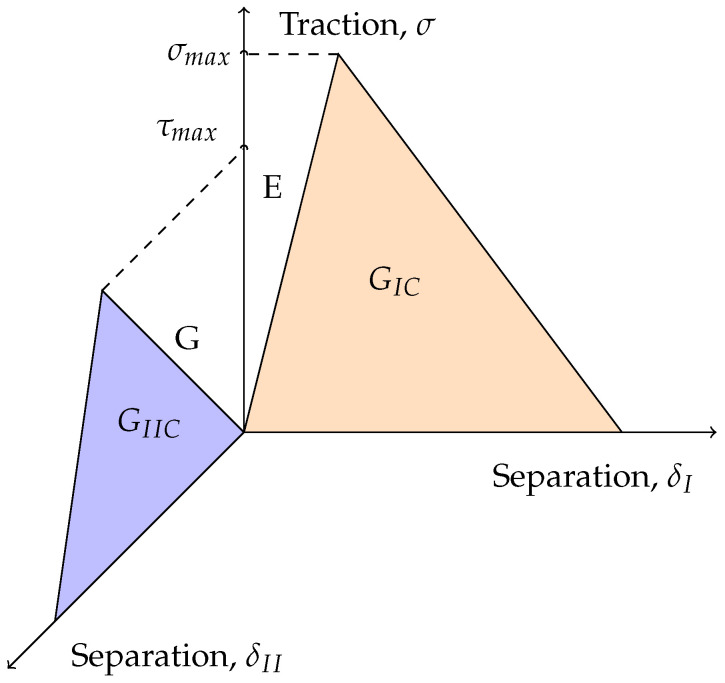
Three-dimensional representation of the fracture envelope of an adhesive, using triangular traction–separation laws. Pure mode I (orange) and pure mode II (blue).

This material behaviour can be defined using the following properties: normal, E, and tangential, G, stiffness values; maximum stresses under normal, $\sigma_{max}$, and tangential, $\tau_{max}$, loading conditions; and critical fracture energies in mode I, $G_{IC}$, and mode II, $G_{IIC}$. Generally speaking, these laws can be shown as a graphical representation of the adhesive properties as seen in Figure 6.

Although the tests performed in this study are related to pure modes, due to the complexities associated with the stress states present in joints, especially in the novel mDCB specimen, the concept of mode mixity needs to be first defined. Usually this phenomenon is established by a Power Law (PW), Equation (1), being of linear order in this case.

(1) $$\left(\frac{G_{I}}{G_{IC}}\right)+\left(\frac{G_{II}}{G_{IIC}}\right)=1$$

#### 2.1.4. Material Properties

In total, two different structural adhesives were used in order to represent different ranges of adhesive’s application and to better highlight the geometrical implications of these characteristics on the overall specimen behaviour.

The first adhesive under consideration is of brittle nature, supplied in a two-component formulation, curing at room temperature and presenting high strength and rigidity. According to its datasheet, when fully cured, the adhesive will have excellent performance at high temperatures and good chemical resistance.

The second adhesive is also strong and stiff, but presents higher ductility and toughness than the previous one. It is a one-part, heat-cured epoxy resin that is compatible with induction curing and can be used in place of brazing, welding, and riveting in high-stress applications. Its key unique feature is improved electrical conductivity and ductility, which are the result of an aluminium filler that the manufacturer added to the adhesive formulation.

The proposed adhesives were mechanically characterised to obtain the necessary elastic and cohesive properties for this simulation: the Young’s modulus, *E*, and shear modulus, *G*; failure tensile strength, $\sigma_{f}$, and failure shear strength, $\tau_{f}$; and finally, critical fracture energies in mode I, $G_{IC}$, and mode II, $G_{IIC}$. An elastic, isotropic behaviour, was assumed for the high strength steel substrates, defined solely by the Young’s modulus and the Poisson’s ratio values.

The properties of each adhesive studied used as the input values for the cohesive zone models (CZM) are listed in Table 1.

#### 2.1.5. Boundary Conditions

Correctly defining the boundary conditions of a model means being as close as possible to the real problem which, in this case, corresponds to the real testing conditions of these two specimens. In the specific case of this unified specimen, each adhesive layer is tested sequentially. This can be identified in relation to Figure 4 by understanding the boundary conditions (BC) that define each test. A summary of their attributes is presented in Table 2.

In the mode I test, all conditions are active, the mDCB test is comprised of BC1, a double support associated with the fixation pin that restricts translation in the *x*- and *y*-directions; BC2, the vertical loading displacement ($u_{y,I}$) applied through a loading pin to the bottom substrate of the ELS, which, at this instance is acting as part of the upper substrate of the mDCB test; and BC3, the moving clamp that, in this stage, solely restricts the rotation of the clamped end of the specimen—linked with the need for a clamping tool in the ELS test.

After the mDCB test ends and its adhesive layer completely fails, the bottom substrate and adhesive layer are removed from the numerical simulation, the ELS specimen can be recognised and the mode II test starts. At this time only two of the boundary conditions are active: BC2, the vertical loading displacement ($u_{y,II}$) applied to the bottom substrate of the ELS through the loading pin; and BC3, the moving clamp that now restricts both the vertical translation and the rotation of the clamped end of the specimen.

Additionally, the ELS specimen, either when considering the isolated test or when it is present in the combined mDCB test, presents the risk of interpenetration of elements in the unbounded region, deviating from the real phenomenon. Moreover, when considering an actual experimental test, the presence of friction at that same region is a common concern. To avoid these phenomena, a standard surface-to-surface contact interaction, with frictionless plus hard contact properties, was specified between the lower and upper unbonded substrate faces by adding a wire structure called *PTFE* (Teflon^®^) that simulated these contact conditions—as is done in experimental tests where the use of this material presents a similar purpose.

### 2.2. Analysed Conditions

To understand how changes in the specimen’s geometry affect the characterisation capability of this configuration, a simple analysis of the load–displacement (*P*-δ) curves is not sufficient.

Using data reduction schemes to interpret the *P*-δ curves results in the formulation of the R-curves, from which the fracture toughness can be extracted. Using these methods to experimentally obtain the fracture properties of the materials used in the numerical simulations is a common practice.

As such, the same methods can be used to validate the numerical simulations. By applying these procedures to the numerical load–displacement (*P*-δ) curves and extracting the related fracture toughness’, it becomes possible to compare them against the material’s input reference values, thus finishing the validation cycle.

This method not only validates the model’s capability to reproduce the real phenomenon, but also the effect of changes in the geometry to the characterisation capability of the specimen. This becomes even more important when considering the novel mDCB specimen, since its behaviour is mostly unknown, but closely relatable with the standard DCB specimen. The closer the model behaves through the same data reduction scheme, the closer it becomes to the desired stage, the mDCB test characterising pure mode I fracture. However, a proper custom data reduction scheme is necessary due to the complexity of this specimen.

The numerical studies conducted in this work start by analysing the behaviour changes in the ELS specimen as a function of its dimensions, and afterwards, assessing the limitations of this test associated with the existence of a fracture process zone (FPZ) and the clamp tool. Finally a study of the geometry of the novel modified DCB test is carried out as a whole but focusing on the influence of the presence of an mostly inactive adhesive layer on the top composed beam, i.e., the ELS specimen. These changes on the mode I characterisation process were compared against a standard DCB test as a reference.

#### 2.2.1. ELS Test

#### Parametric Study

Both Figure 2, showing the isolated ELS, and Figure 4, showing the unified specimen, present the relevant geometric parameters used to design the end-loaded split specimen: $L_{\mathrm{ELS}}$ is the length of the specimen, $L_{clamp}$ the clamped length, $d_{\mathrm{ELS}}$ the mid span length of the specimen, i.e., the useful adhesive, ${a_{0}}_{\mathrm{ELS}}$ the initial crack length, *t* is the thickness of the adhesive layer, $h_{\mathrm{ELS}}$ the height of the substrate and *b* is the width of the specimen.

In Table 3, the initial geometrical properties of the ELS specimen modelled are presented. These dimensions were based on the double cantilever beam specimen already used in the ENF test for mode II adhesive characterisation and will serve as the base specimen used in the numerical optimisation of the ELS specimen geometry.

Other than the adhesive thickness, *t*, substrate width, *b*, and clamped length, $L_{Clamp}$, which remain fixed, all ELS dimensions were sequentially analysed to reduce the total work load. This will be conducted by holding the base values as references, and exploring alterations that improve the overall performance of the specimen.

With this in mind, firstly, the initial crack length is tested, where, for both adhesives, the ${a_{0}}_{\mathrm{ELS}}$ was varied in 20 mm increments: 80 mm, 100 mm and 120 mm—for the brittle adhesive; and 40 mm, 60 mm and 80 mm—for the tough adhesive. Followed by the total specimen length, where $L_{\mathrm{ELS}}$ was varied in 30 mm increments: 230 mm, 260 mm and 290 mm. Next came the specimen height, which for both adhesives $h_{\mathrm{ELS}}$ was varied in 3.7 mm increments: 16.4 mm, 12.7 mm and 9.0 mm. In the end, to test the importance of the useful adhesive length, $d_{\mathrm{ELS}}$, two combinations of ${a_{0}}_{\mathrm{ELS}}$ and $L_{\mathrm{ELS}}$ were modelled for each adhesive, giving constant mid span length values of $d_{\mathrm{ELS}}=$ 90 mm for the brittle adhesive; and 130 mm for the tough adhesive.

#### Fracture Process Zone Analyses

As mentioned before, a fracture process zone (FPZ) is developed ahead of the major crack tip due to plasticization and the nucleation of many micro-cracks. The stability of the end-loaded split test thus depends on the complete formation of the full FPZ, implying that the aforementioned mid-span length must be large enough for this phenomenon to occur. Since this test has the particularity of using a clamping tool, an additional study was conducted to understand whether the FPZ could be affected by the clamped region, influencing the stability of the test, as some studies have already shown.

To better acknowledge the relationship between this mid span length and the formation of the FPZ, this study compares the development of this FPZ by means of the lengths of these regions, $L_{\mathrm{FPZ}}$, against the evolution of the equivalent crack, $a_{eq}$, extracted from the ELS data reduction scheme later presented, and the effective growth of the crack, *a*, in the specimen.

This analysis makes use of the damage features of Abaqus^®^, where the damage evolution of the adhesive is based on that used for ductile metals, which presupposes that damage manifests as a progressive degradation in material stiffness that ultimately results in material collapse. A damage initiation criterion must be utilised in conjunction with the damage evolution. After damage initiation, this criterion uses mesh-independent measurements, either plastic displacement or physical energy dissipation, to control damage evolution. It also considers the combined role of different damage mechanisms acting simultaneously on the same material, offers options for describing how each mechanism contributes to the overall degradation of the material and provides options for what happens in the event of failure, such as the removal of elements from the mesh [47].

**Figure 7:**
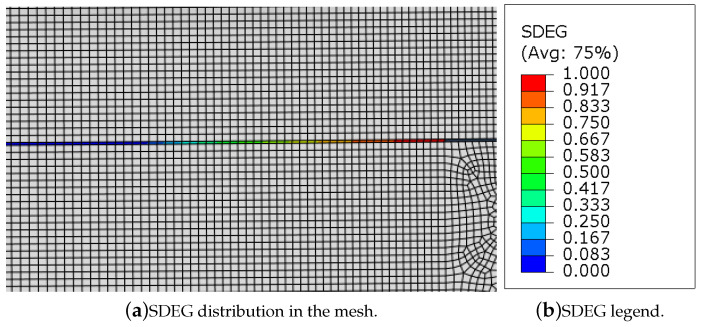
Stiffness degradation, SDEG, parameter in Abaqus^®^.

The length, $L_{\mathrm{FPZ}}$, and development, *a*, of the fracture process zone were then computed using the Abaqus’ damage parameter SDEG (Stiffness DEGradation), studied along a path created through the adhesive layer, measuring the state of damage of each element that it crosses, as an overall scalar stiffness degradation. When this parameter is zero the element is not damaged but when it reaches its maximum value, 1, the element is completely degraded and is deleted, meaning that the crack started to propagate, i.e., *a* increased. In Figure 7a, it is possible to observe the crack tip of the ELS specimen and the correspondent damage region ahead of it, which is coloured following a certain damage distribution, as shown in Figure 7b. Therefore, the length of the region where the SDEG parameter is higher than zero is the length of the FPZ, $L_{\mathrm{FPZ}}$. This parameter is related to another one, STATUS, which provides, as indicated by its name, the status of the element, being 1 if the element is active and 0 if the element is not. Once the SDEG parameter reaches 1, the STATUS parameter starts to identify elements that become active and eliminates them from the adhesive layer elements.

As aforementioned, the SDEG damage parameter was used to estimate the fracture process zone development and, consequently, its length, $L_{\mathrm{FPZ}}$, along with the adhesive layer. In terms of the crack propagation, *a*, it was computed using the STATUS parameter, allowing to identify when an element has reached its maximum damage state and to delete it from the adhesive layer increasing the overall crack length. Both these parameters were measured in terms of true distance from the start of the chosen path, i.e., the crack tip. The equivalent crack evolution was determined using the ELS data reduction formulation, and it is also represented in terms of true distance from the initial crack tip. For both adhesive, two conditions were studied, by changing the total length of the specimen, $L_{\mathrm{ELS}}$, from 290 mm to 230 mm.

#### 2.2.2. Modified DCB Test

#### Parametric Study

Figure 4, the unified specimen, presents the relevant geometric parameters used to design the modified double cantilever beam specimen: $L_{\mathrm{mDCB}}$ is the length this specimen; $d_{\mathrm{mDCB}}$ the mid span length of the specimen, i.e., the useful adhesive; ${a_{0}}_{\mathrm{mDCB}}$ the initial crack length of the mDCB specimen; *t* is the thickness of the adhesive layer; $h_{\mathrm{mDCB}}$ the height of the bottom bar of the mDCB specimen; and *b* is the width of the specimen. Additionally, the dimensions associated with the top bar of the mDCB specimen are also important, it being an ELS specimen composed of two bars of height $h_{\mathrm{ELS}}$ (the adhesive thickness, *t*, will be ignored); and ${a_{0}}_{\mathrm{ELS}}$ the initial crack length of the ELS specimen, these were also changed and presented great influence on the results obtained. The adhesive thickness, *t*, substrate width, *b*, and clamped length, $L_{Clamp}$, remained the same as for the ELS specimen, Table 3.

The base geometry for the mDCB test specimen is described in Table 4.

Since the standard DCB test was used as a reference to study the mDCB specimen, Figure 1 presents the relevant geometric parameters used to design the double cantilever beam specimen: $L_{\mathrm{DCB}}$ is the length this specimen; $d_{\mathrm{DCB}}$ the mid span length of the specimen, i.e., the useful adhesive; ${a_{0}}_{\mathrm{DCB}}$ the initial crack length of the mDCB specimen; *t* is the thickness of the adhesive layer; $h_{\mathrm{DCB}}$ the height of the substrates; and *b* is the width of the specimen. The adhesive thickness, *t*, and substrate width, *b*, remained the same as for the mDCB specimen, Table 3.

The base geometry for the DCB test specimen is described in Table 5.

Once more, other than the adhesive thickness, *t*, substrate width, *b*, and clamped length, $L_{Clamp}$, which remain fixed, all ELS and mDCB dimensions will be analysed. This will be conducted by holding the base values and the findings of the ELS study as references, and exploring alterations that improve the overall performance of the specimen. The DCB specimens used as a references through out the study were tested with the base dimensions, only changing the initial crack length, ${a_{0}}_{\mathrm{DCB}}$, to better compare the specimen’s stiffness against the mDCB configurations.

With this in mind, firstly, the relationship between the initial crack length of the ELS and mDCB adhesive layers was the main parameter to be tested. As such, for each adhesive, initially ${a_{0}}_{\mathrm{mDCB}}$ was fixed at the base value defined in Table 4, and ${a_{0}}_{\mathrm{ELS}}$ was varied from ${a_{0}}_{\mathrm{ELS}}>{a_{0}}_{\mathrm{mDCB}}$ to ${a_{0}}_{\mathrm{ELS}}\leq {a_{0}}_{\mathrm{mDCB}}$. Then, taking the findings from the previous analysis, for each adhesive a deeper study on the best [${a_{0}}_{\mathrm{mDCB}}$; ${a_{0}}_{\mathrm{ELS}}$] configurations was conducted, reaching a final recommendation of crack length relations for each adhesive type.

Finally, several combinations of $h_{\mathrm{mDCB}}$ and $h_{\mathrm{ELS}}$ were tested, considering also the previously defined specimen recommendations for the other dimensions.

#### 2.2.3. Data Reduction Methods

The compliance-based beam method (CBBM) was used to analyse the load–displacement curves obtained in each test resulting in the definition of the R-curves for each fracture mode and test type. This data reduction scheme has the advantage of dispensing with a direct crack length determination, as well as taking into account the fracture process zone [6].

#### Mode I—CBBM for DCB Test

Considering this method, fracture energy for mode I for a standard DCB is given by Equation (2) [6]:

(2) $$G_{I}=\frac{6P^{2}}{b^{2}h_{I}}\left(\frac{2a_{eqI}^{2}}{h_{I}^{2}E_{fI}}+\frac{1}{5G_{13}}\right)$$

where *P* is the load applied, *b* the specimen width, $h_{I}$ the thickness of the substrates, $G_{13}$ their shear modulus, $a_{eqI}$ the equivalent crack length and $E_{fI}$ the corrected flexural modulus. This last parameter is needed since this method does not take into account stress concentration and the substrates’ rotation near the crack tip [6]. This reduction method was used initially in both the DCB test and the mDCB test in order to compare their differences, even when considering that the analytical formulation does not consider the geometry associated with the modified DCB. All this is due to the introduction of an ELS adhesive layer in the upper substrate of the modified double cantilever beam specimen—which, in the standard DCB test is actually a solid beam—making the stress distribution more complex and non-symmetric.

However, since ideally, the mDCB test needed to be handled as an asymmetric double cantilever beam, for which there is currently no crack independent data reduction scheme, a novel mDCB CBBM was formulated.

#### Mode I—CBBM for mDCB Test

In order to correctly define the behaviour of the novel mDCB specimen, which does not yet account for a data reduction method, a custom variation of CBBM was devised. The method’s full formulation is explained in Appendix A, resulting in the following expression for the fracture energy for mode I of the mDCB specimen (Equation (3)):

(3) $$G_{I}=\frac{P^{2}}{2b}\left(\left(\frac{12a_{eqI}^{2}}{E_{fI}bh_{I}^{3}}+\frac{6}{5G_{I}bh_{I}}\right)+\left(\frac{3a_{eqI}^{2}}{2E_{fII}bh_{II}^{3}}+\frac{3}{5G_{II}bh_{II}}\right)\right)$$

being *P* the applied load, *b* the specimen width, $h_{I}$ the thickness of the mDCB substrate, $h_{II}$ the thickness of the ELS substrates, $G_{I}=G_{II}=G_{13}$ their shear modulus, $a_{eqI}$ the mDCB equivalent crack length, and $E_{fI}$/$E_{fII}$ the corrected flexural moduli for the mode I and mode II components of the formulation, respectively. These last parameters take again into account the stress concentration and the substrates’ rotation near the crack tip in a similar way as defined for a standard DCB [6] and ELS [11]. This reduction method was used for the mDCB test.

Additionally, for this method to work properly, the initial compliance ratio (*r*—required for the equation presented in Appendix A) must be defined. For this purpose, the rotation of each loading point was tracked and extracted from the model, resulting in a precise definition of the contributions of each beam to the total compliance of the specimen.

#### Mode II—CBBM for ELS Test

Similarly, to obtain the energy release rate in mode II, a similar Equation (Equation (4)) can be used [11]:

(4) $$G_{II}=\frac{9P^{2}a_{eqII}^{2}}{4b^{2}E_{fII}h_{II}^{3}}F_{2}$$

where for both equations *P* is the load applied, *b* the specimen width, $h_{II}$ the thickness of the substrates, $a_{eqII}$ the equivalent crack length, $E_{fII}$ the corrected flexural modulus, and $F_{2}$ is a large displacements correction factor. This reduction method was used for the ELS test.

## 3. Numerical Results and Discussion

The results of the parametric study on specimen’s geometry and the evaluation of its effect on specimen performance and the extracted data are presented in this section. Some relevant dimensions for the end-loaded split and unified specimen were studied in order to compute behaviour changes of this test, whether by analysing the load–displacement curves (*P*-δ curves) or the resultant R-curves, computed using the compliance based beam method (CBBM) for each test. The DCB test were treated as in [6], the mDCB through its own novel formulation, and the ELS as in [11].

In order to be able to validate the proposed testing methodology for a wide range of adhesive applications, two different adhesives were considered. First, the study mentioned above was carried out for a more brittle adhesive, and then the same approach was applied to a tougher adhesive, employing cohesive zone laws based on the mechanical properties listed in the previous section. The results for each adhesive will be presented side by side for comparison purposes.

### 3.1. End-Loaded Split (ELS) Test Specimen

Although the works reported by Pérez-Galmés et al. [21] and Blackman et al. [22] describe some of the criteria necessary to design ELS joints and the several guidelines for their dimensions, their work focuses on adhesive joints with composite substrates. Therefore, their results are not applicable to the case of this study, where steel substrates are used.

As such, the key dimensions of steel ELS adhesive joints were analysed in this numerical study. The first parameter considered in the study was the initial crack length ${a_{0}}_{\mathrm{ELS}}$, then the total length of the specimen, $L_{\mathrm{ELS}}$, and finally the substrates’ thickness, $h_{\mathrm{ELS}}$. Furthermore, a complementary study on the development of the fracture process zone, $L_{\mathrm{FPZ}}$, in this type of specimens was carried out. Some other considerations about other parameters directly related to this main ones were also made.

#### 3.1.1. Parametric Study

Since similar behaviours for each adhesive were identified, the conclusions drawn will not refer to a specific adhesive but to the analysed phenomenon as a whole.

##### Initial Crack Length—${a_{0}}_{\mathrm{ELS}}$

**Figure 8:**
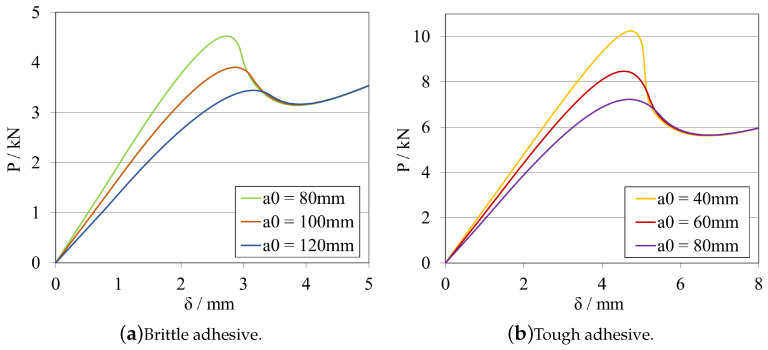
*P*-δ curves related to the ${a_{0}}_{\mathrm{ELS}}$ study.

A study about the influence of changing the initial crack length size was carried out for the model presented previously. The obtained load–displacement curves are shown in Figure 8a, for the brittle adhesive, and in Figure 8b, for the tough adhesive.

**Figure 9:**
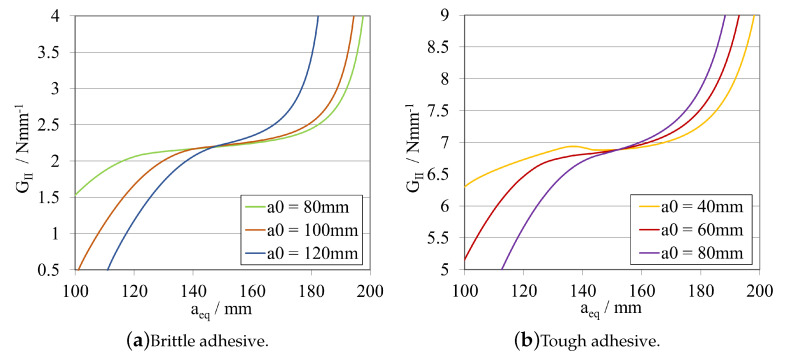
R-curves related to the ${a_{0}}_{\mathrm{ELS}}$ study.

The corresponding R-curves and $G_{IIC}$ values, computed using CBBM, are presented in Figure 9a, for the brittle adhesive, and in Figure 9b, for the tough adhesive.

At this stage, it is possible to conclude that both adhesives exhibit similar behaviour in terms of *P*-δ and R-curves, even considering that they present different ${a_{0}}_{\mathrm{ELS}}$ ranges. This behaviour is related to the fact that the useful adhesive length required for the full development of the fracture process zone in each adhesive is different. For brittle adhesives, a reduced useful adhesive length is needed for this development. However, the length needed for this phenomenon to occur is evidently higher in the case of more tough/ductile adhesives.

As a result, to enable stable crack propagation, each adhesive needs different crack length ranges, larger for the brittle one and smaller for the tough adhesive. Nevertheless, and for both cases, by decreasing ${a_{0}}_{\mathrm{ELS}}$, a more pronounced plateau can be obtained, providing a more direct extraction of the mode II fracture energy release rate.

However, when decreasing the initial crack, ${a_{0}}_{\mathrm{ELS}}$, for the same specimen length and clamping conditions, the failure mechanism becomes more brittle/unstable. This is due to the maximum flexural moment ($\approx P_{max}\times L_{\mathrm{ELS}}$) being higher for lower ${a_{0}}_{\mathrm{ELS}}$ values. As the crack starts to propagate, due to the much higher kinetic energy stored prior to crack propagation, the phenomenon becomes unstable, resulting in a steeper transition from the highest peak to the lowest peak of the *P*-δ curve (Figure 8), associated with a very small displacement of the loading pin. This might lead to problems related with unstable crack propagation when being carried out experimentally. Additionally, depending on the capabilities of the data acquisition systems, it could be challenging to gather enough points to compute a reliable R-curves when sudden failures occur. In order to provide a geometric guide for designing these specimens, stability criteria that relates the ratio between the initial crack tip and the total length of the specimen have been developed for composite materials establishing a ratio of ${a_{0}}_{\mathrm{ELS}}/L_{\mathrm{ELS}}\geq 0.55$.

The initial slope of the *P*-δ curve, can be understood as the stiffness of the specimen prior to being damaged. As expected, this slope is different for each adhesive, as the stiffness of the specimen is a function not only of the substrates but also the adhesive joint properties and dimensions. Furthermore, it is also reasonable to conclude that, by increasing the initial crack tip size, the specimen’s stiffness is directly influenced. The specimen becomes less stiff, since a longer crack tip provides less structural integrity to the specimen, acting as a larger defect.

In order to explore the effects of altering the overall length $L_{\mathrm{ELS}}$ of the ELS specimen, one midway value of ${a_{0}}_{\mathrm{ELS}}$ was selected for each adhesive, taking into account the aforementioned factors: 100 mm for the brittle adhesive, and 60 mm for the tough adhesive.

##### Total Length—$L_{\mathrm{ELS}}$

**Figure 10:**
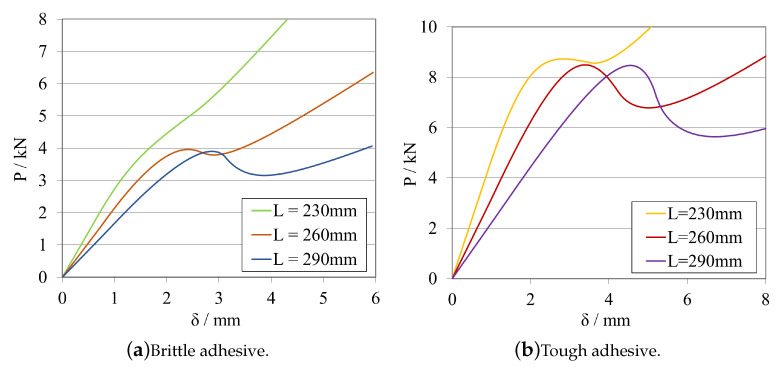
*P*-δ curves related to the $L_{\mathrm{ELS}}$ study.

In this subsection, a study on the influence of changing the total length of the specimen was carried out. The obtained load–displacement curves are shown in Figure 10a, for the brittle adhesive, and in Figure 10b, for the tough adhesive.

**Figure 11:**
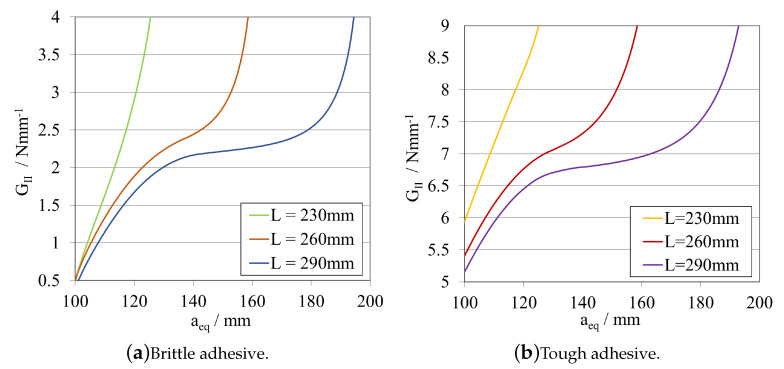
R-curves related to the $L_{\mathrm{ELS}}$ study.

Each R-curve and $G_{IIC}$ value, computed using CBBM, is presented in Figure 11a, for the brittle adhesive, and in Figure 11b, for the tough adhesive.

From Figure 10 and Figure 11a, for the brittle adhesive, and Figure 10 and Figure 11b, for the tough adhesive, the influence of the 30 mm decrements on the fracture mechanisms associated with crack propagation recognisable.

Taking for example, a double cantilever beam loaded with *P* on the free end, the displacement is given by Equation (5):

(5) $$\delta =\frac{PL^{3}}{3EI}$$

where *E* is the Young’s modulus of the beam’s material and *I* the second moment of inertia. The stiffness of the beam is then given by Equation (6).

(6) $$K=\frac{P}{\delta }\Leftrightarrow K=\frac{3EI}{L^{3}}$$

Since the overall stiffness of the beam is influenced by the power of 3 of its total length, by decreasing *L*, there is a substantial increase in *K*. Not only is there less space for the crack to fully propagate, but the beam itself becomes excessively stiff, making it impossible to attain a stable propagation, even if using a displacement controlled test.

Given that a general conclusion is that the larger the total length of the specimen, the more favourable the stable propagation conditions that arise. However, this might not be feasible in many practical cases since it means that exceedingly large specimens might need to be manufactured, causing possible constraints with not only the manufacturing process but also with the preparation of adequate testing setups and load cell capacity.

Therefore, the $L_{\mathrm{ELS}}$ was kept constant and equal to the original base value, 290 mm, for studying the influence of the substrate’s height in *P*-δ curves and R-curves. The initial crack lengths were maintained in the next step.

##### Substrate Height—$h_{\mathrm{ELS}}$

**Figure 12:**
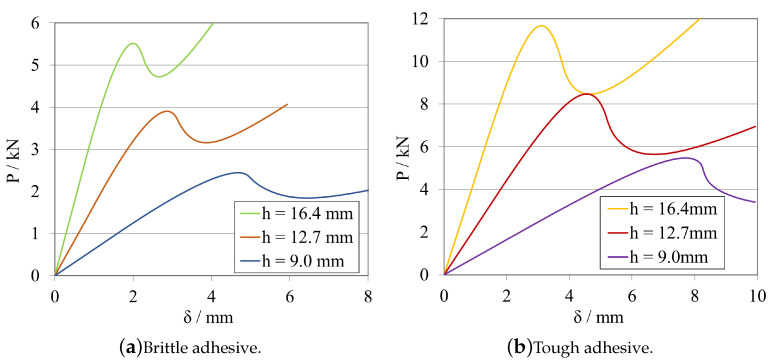
*P*-δ curves related to the *h* study.

**Figure 13:**
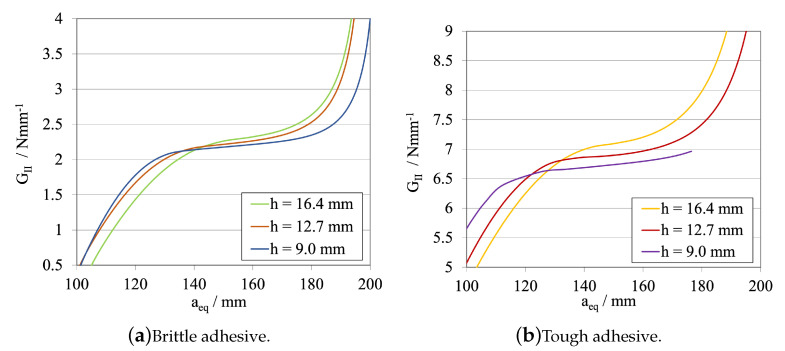
R-curves related to the *h* study.

Figure 12 and Figure 13a, for the brittle adhesive, and Figure 12 and Figure 13b, for the tough adhesive, present a study of the substrate’s height influence in both the load–displacement curves and R-curves, respectively.

Similarly to what was observed during the analysis of the total length of the specimen, there is a direct, non-adhesive-dependent relationship between the increase in substrate thickness and the increase in stiffness of the specimen, which is also explained using the cantilever beam example. In this case, $h_{\mathrm{ELS}}$ directly influences the second moment of inertia, considering the rectangular shape of the cross section area of the specimens, as follows—Equation (7).

(7) $$I=\frac{bh^{3}}{12}$$

which, in terms of stiffness, *K*, reflects similarly to what is presented in Equation (8).

(8) $$K=\frac{Ebh^{3}}{4PL^{3}}$$

Having a weight of power 3 on the stiffness of the substrate, the substrate thickness can be considered as a relevant parameter when designing ELS specimens. When analysing the corresponding R-curves, it is possible to observe that there are not significant changes in their shape, since the CBBM formulation presupposes a small influence of this variable for the computation of the fracture energy release rate in mode II, $G_{IIC}$. However, greatly increasing the specimen stiffness, as it happens in the last two studies, may introduce additional stability problems when performing theses tests experimentally. Nevertheless, excessive flexibility is also prone to result in large deflections which, when considering plasticity phenomena, leads to substrate yielding, which is undesirable and may result in need of use of ultra-high-strength steel substrates. Being so, a middle value was maintained and kept constant to continue this study, $h_{\mathrm{ELS}}=$ 12.7 mm.

##### Mid Span Length—*d*

The following plots compare different combinations of the total length of the specimen and the initial crack tip length, for the same mid span length, denoted as $d_{\mathrm{ELS}}$. The same mid span length is understood as the distance between the crack tip and the clamping tool, i.e., the useful adhesive, which can be obtained by different combinations of $L_{\mathrm{ELS}}$ and ${a_{0}}_{\mathrm{ELS}}$, as mentioned.

**Figure 14:**
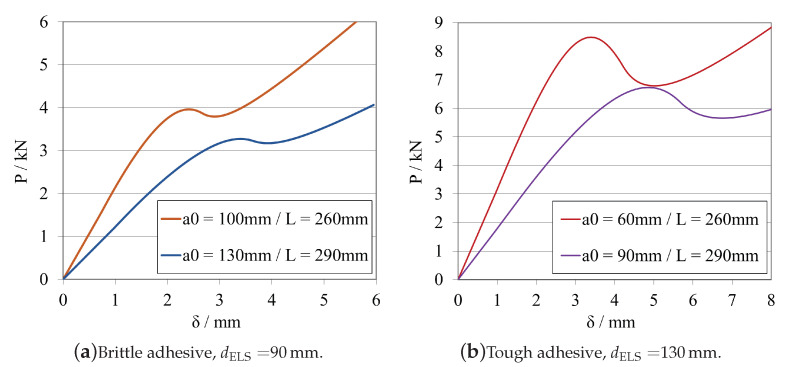
*P*-δ curves related to different combinations of ${a_{0}}_{\mathrm{ELS}}$ and $L_{\mathrm{ELS}}$, to attain the same $d_{\mathrm{ELS}}$.

**Figure 15:**
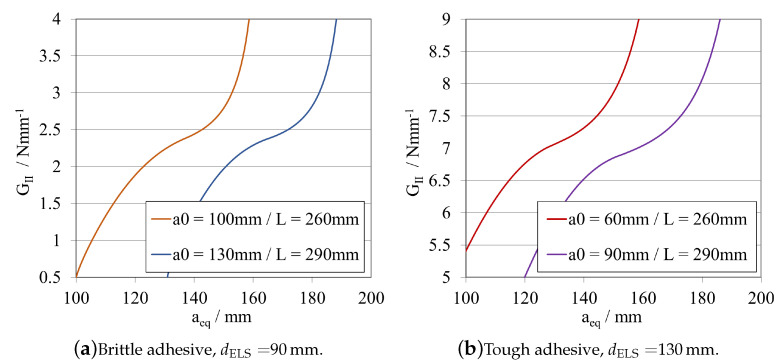
R-curves related to different combinations of ${a_{0}}_{\mathrm{ELS}}$ and $L_{\mathrm{ELS}}$, to attain the same $d_{\mathrm{ELS}}$.

In Figure 14a,b, the load–displacement curves presented for $d_{\mathrm{ELS}}$ are equal to 90 mm, for the brittle adhesive, and 130 mm, for the tough adhesive, both obtained by different combinations of ${a_{0}}_{\mathrm{ELS}}$ and $L_{\mathrm{ELS}}$. Sequentially, the corresponding R-curves, for the same case are presented on Figure 15a,b, respectively.

When analysing the *P*-δ curve, it is evident that there is a significant variation in stiffness, yet there is no apparent change when analysing the R-curve, where both curves have exactly the same shape, but different $a_{eq}$, related to the different initial crack lengths. This then postulates that the R-curve’s shape depends mostly on the adhesive length available for the development of the FPZ.

The previous affirmations are supported, firstly, by the fact that decreasing the total length of the specimen, immediately increases the stiffness by a power of 3, as demonstrated in Equation (6). Furthermore, secondly, by the fact that both variables, ${a_{0}}_{\mathrm{ELS}}$ and $L_{\mathrm{ELS}}$ have the same weight in the $G_{IIC}$ formulation, related to the same exponent.

When selecting the mid span length, it is important to understand that this is the length available for crack propagation, which can be potentially more or less stable, depending on the geometry of the specimen. It was shown that having both a bigger ${a_{0}}_{\mathrm{ELS}}$ and $L_{\mathrm{ELS}}$ propitiates a more stable crack propagation. Consequently, in this case, where the mid span length is kept constant, it is preferable to combine having both a bigger total length and initial crack length, since the specimen presents less overall stiffness and a more progressive crack propagation path.

#### 3.1.2. Fracture Process Zone (FPZ) Study

This study compares the evolution of the following three variables: fracture process zone length, $L_{\mathrm{FPZ}}$; equivalent crack, $a_{eq}$; and actual crack propagation, *a*; relating these variables with the displacement applied to the specimen. The vertical axis of the plot goes from the crack tip to the clamp tool, measuring the variable progression through the whole adhesive layer.

#### Brittle Adhesive

**Figure 16:**
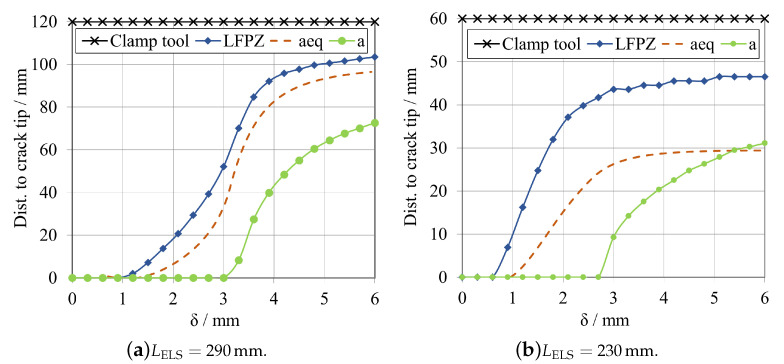
Study of $L_{\mathrm{FPZ}}$, $a_{eq}$ and *a* for the brittle adhesive, with ${a_{0}}_{\mathrm{ELS}}=$100mm.

For the brittle adhesive, two cases where considered, Figure 16a for a $L_{\mathrm{ELS}}=$ 290 mm, and Figure 16b for a $L_{\mathrm{ELS}}=$ 230 mm, both with an ${a_{0}}_{\mathrm{ELS}}$ of 100 mm.

For the first case, related to Figure 16a, it is possible to observe that none of these parameters reached the area of influence of the clamped area prior to propagation, defined by Pérez-Galmés et al. [21] as approximately the 10mm near the clamping tool. This was expected since for this specimen design, it was shown in Figure 10 and Figure 11a that it is possible to attain a secure plateau for the $G_{IIC}$ extraction before any interaction with this boundary condition. Similarly to the $L_{\mathrm{FPZ}}$ study performed by Blackman et al. [22], a proper FPZ development can be characterised by crack propagation beginning while $L_{\mathrm{FPZ}}$ is still developing smoothly and is far from the clamp’s area of influence.

As mentioned in Section 2.2.3, CBBM accounts for the effect of the formation of the fracture process zone, so the evolution of the equivalent crack, $a_{eq}$ should be sufficient to determine a sufficient mid-span length, $d_{\mathrm{ELS}}$, to have stable propagation. However, the fracture process zone length is more significant than what the $a_{eq}$ accounts for, which means that this length should be considered in the specimen’s design.

On the other hand, it is possible to see that the FPZ starts growing before the other parameters, which is also expected since the FPZ is a representation of the damage being accumulated ahead of the crack tip. In comparison, the real crack, *a*, only starts propagating later due to the fact that the damage variable, SDEG, only first reaches 1 around 3.2 mm of displacement. The difference between the starting point of the fracture process zone length and the equivalent crack has to do, as previously mentioned, with the fact that the length of the FPZ considers damage differently, using the SDEG parameter whilst the $a_{eq}$ is formulated following the naturally indirect CBBM propositions, that consider this length as a point somewhere inside the FPZ, so in between the $L_{\mathrm{FPZ}}$ and the actual *a*.

Identifying conditions where the evolution of these three parameters is hindered or remains constant and stops developing is also feasible. This is due to compressive stresses induced by the clamp tool, which restrict the movement of the specimen in that region. As a result, a barrier is formed preventing the amount of adhesive plastic deformation necessary for proper crack propagation [21]. Observing the plot present in Figure 16b, the $L_{\mathrm{ELS}}=$ 230 mm configuration shows precisely this phenomenon, making it unsuitable since it does not present proper crack propagation, as seen in both Figure 10 and Figure 11a.

It is immediately noticeable that none of the parameters smoothly develop towards the clamped region, and start to stabilise even before the actual crack, *a*, starts to develop. As mentioned before, the clamping tool induces compressive stresses on the adhesive, restraining its plastic deformation and hindering the damage evolution, sometimes not even allowing a failure mechanism to take place. Even if the clamping tool allows movement in the *x*-direction, the fixed restriction applied to the vicinity of the clamped zone makes so that any kind of relative displacement between the specimen’s substrates does not occur. This relative movement would inherently induce damage in the adhesive, however, it is restricted in the close vicinity of the clamp tool, explaining why the damage-related parameters ($L_{\mathrm{FPZ}}$ and $a_{eq}$) never reached the clamp tool and stopped prematurely. For the case of this model, where compressive stresses bear a significant influence in determining the end of the development of FPZ, the instability of the test is related to the fact that the FPZ is not long enough to allow stable crack propagation. This means that even if the clamped region is not reached, the specimen’s geometry is still not propitious for the crack to fully propagate and to attain a plateau in the R-curve.

This means that the length of the fracture process zone is a relevant parameter in designing these specimens, related also with the previously studied mid span length, $d_{\mathrm{ELS}}$, which must be long enough to fully develop the FPZ. Some authors have proposed analytical formulae to determine this length, but they usually apply to specific cases different from this one, such as composites.

#### Tough Adhesive

**Figure 17:**
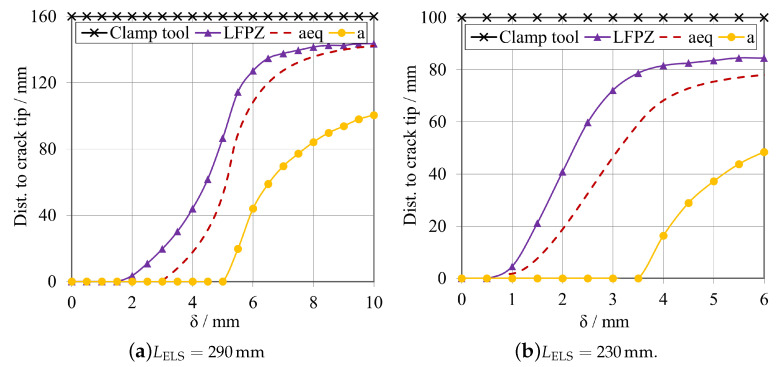
Study of $L_{\mathrm{FPZ}}$, $a_{eq}$ and *a*, for the tough adhesive, with ${a_{0}}_{\mathrm{ELS}}=$60 mm.

The study relating the mid span length and the evolution and growth of the fracture process zone to the equivalent crack and the actual crack was also repeated for the tough adhesive using the same procedure. For this adhesive, two cases were considered, Figure 17a for a $L_{\mathrm{ELS}}=$ 290 mm, and Figure 17b for a $L_{\mathrm{ELS}}=$ 230 mm, both with an ${a_{0}}_{\mathrm{ELS}}$ of 60 mm.

As expected, once more, none of the parameters under analysis reached the clamp’s area of influence, defined by Pérez-Galmés et al. [21]. For the case presented in Figure 17a, a plateau is achieved. The fracture process zone length is more significant than the equivalent crack length evolution, but $a_{eq}$ follows $L_{\mathrm{FPZ}}$ quiet closely, making it a reliable parameter to track the crack evolution present in the adhesives used in this study. In this case, it is also possible to identify a set of conditions where the evolution of these three parameters stops and remains constant. Again, this is because the clamping tool’s compressive stresses act as a barrier to the adhesive’s plastic deformation and significantly restrict the crack’s ability to propagate. Furthermore, similarly to the previous adhesive, a proper FPZ development was obtained, when considering the study performed by Blackman et al. [22] as a reference.

Similarly to what was done for the brittle adhesive, a study of the evolution of these parameters was conducted for a different specimen’s geometry, represented in Figure 17b, for the $L_{\mathrm{ELS}}=$ 230 mm, in order to demonstrate the same phenomenon occured. As it happened for the first adhesive, although it was expected that some of the parameters under analysis could progress and reach the clamping tool, since the specimen is shorter and the mid span length is consequently shorter, this situation was not verified. Although the effective space for crack propagation is indeed shorter, this did not imply that any of the parameters developed under the clamp tool affected the stability of the tests and prevented attaining a plateau for $G_{IIC}$, as seen in Figure 11b. In fact, the reason behind this difficulty in attaining the plateau is related to a lack of a minimum length for proper crack propagation, hindered by the compressive stresses induced by the clamp tool.

### 3.2. Modified Double Cantilever Beam (mDCB) Test Specimen

As mentioned before, a new unified specimen for combined mode I and mode II fracture characterisation was developed. This specimen is to be sequentially loaded, performing firstly a mode I fracture test, a modified double cantilever beam (mDCB) test, and, afterwards, the same load cell would measure the load being transferred solely to the upper part of the specimen, performing a mode II fracture test, on an end-loaded split (ELS) specimen. Since the ELS specimen geometry was already analysed for both adhesives, only the mode I component needs to be parametrically tested as well.

To facilitate interpretation of the graphs, each configuration presents a compact code to identify the case under study in an easy and fast manner, as identified by the following code: “test type”—DCB or mDCB— followed by the values of the parameter under study, in millimetres, “DCB dimension” or “mDCB dimension/ELS dimension”, e.g., $a_{0}$ study with "DCB 80" and "mDCB 80/60", means a DCB with ${a_{0}}_{\mathrm{DCB}}=$ 80 mm and an mDCB with ${a_{0}}_{\mathrm{mDCB}}=$ 80 mm and ${a_{0}}_{\mathrm{ELS}}=$ 60 mm.

#### Parametric Study

To understand the influence that the upper mDCB substrate, corresponding to the ELS specimen, would have on the *P*-δ curves of this test, different configurations were tested focusing specifically on the influence of the ${a_{0}}_{\mathrm{ELS}}$ on the characterisation capabilities of the mDCB specimen.

Following the recommendations of the ELS parametric study, the base dimensions of the mDCB test were established: $L_{\mathrm{ELS}}$ was kept at the original value; $L_{\mathrm{mDCB}}$ was set to allow for $L_{Clamp}$ and still have free space to accommodate the apparatus; ${a_{0}}_{\mathrm{mDCB}}$ was defined as a small value commonly used in the DCB tests; $h_{\mathrm{mDCB}}$ was set at the same value of the standard DCB test; $h_{\mathrm{ELS}}$, was defined at the minimum value tested for the ELS, to better approximate the height of the bottom bar to the upper bar; and finally, the ${a_{0}}_{\mathrm{ELS}}$ was tested for various relations against ${a_{0}}_{\mathrm{mDCB}}$ – larger, equal and smaller. All of these base dimensions can be seen in Table 4.

As previously detailed, standard DCB tests were used as references to which the mDCB test should correlate, therefore, the more similar the curves of both tests are, the better the mDCB characterisation behaviour is—ideally they would be superposed. This relation is more relevant when the crack is growing, meaning that parallel propagation stages between the mDCB and DCB tests are the best way to understand that a good and stable crack propagation is happening, even if the overall value of $G_{IC}$ is either over or under estimated, since a correction could be easily applied.

Having very similar behaviours for each adhesive the conclusions drawn did not refer to a specific adhesive but to the analysed phenomenon as a whole.

##### Initial Crack Length Study—${a_{0}}_{\mathrm{ELS}}>{a_{0}}_{\mathrm{mDCB}}$

**Figure 18:**
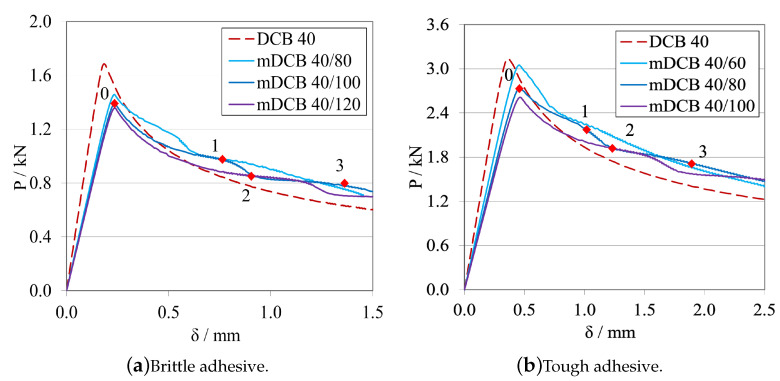
*P*-δ curves related to ${a_{0}}_{\mathrm{ELS}}>{a_{0}}_{\mathrm{mDCB}}$, compared against the correspondent standard DCB. Auxiliary red markers represent the stages of crack evolution for the intermediary dark blue curve.

**Figure 19:**
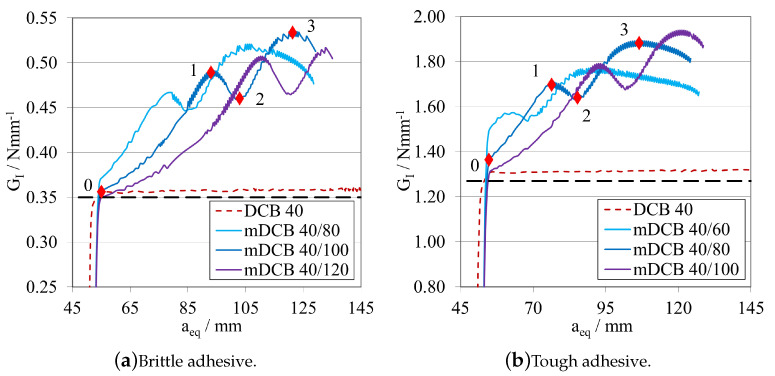
R-curves related to ${a_{0}}_{\mathrm{ELS}}>{a_{0}}_{\mathrm{mDCB}}$, compared against the correspondent standard DCB. Black dashed line represents the input $G_{IC}$ value. Auxiliary red markers represent the stages of crack evolution for the intermediary dark blue curve.

Leaving the ELS parametric study with the notion that having a larger initial crack length, ${a_{0}}_{\mathrm{ELS}}$, was beneficial, made this study progress towards the analysis of the influence of this notion into the mode I specimen. As such the following procedure was defined: ${a_{0}}_{\mathrm{mDCB}}$ was kept constant at 40 mm for both adhesives; and, for each adhesive, three ${a_{0}}_{\mathrm{ELS}}$ were tested, the values in question are present in the plot legend codes. Additionally, a standard DCB with the same ${a_{0}}_{\mathrm{DCB}}$ was tested to define a reference behaviour. The *P*-δ curves and R-curves are presented in Figure 18 and Figure 19a, for the brittle adhesive, and Figure 18 and Figure 19b, for the tough adhesive. These modified DCB results were analysed by the standard DCB CBBM, since the novel approach was not valid while ${a_{0}}_{\mathrm{ELS}}>{a_{0}}_{\mathrm{mDCB}}$.

After interpreting the results for the mode I test, it is possible to state that contrarily to what was defined in the previous parametric study, the use of a large ${a_{0}}_{\mathrm{ELS}}$ is not completely beneficial if its value is higher than ${a_{0}}_{\mathrm{mDCB}}$. Presenting a strange waviness phenomena, the values of $G_{IC}$ seen in Figure 19 are both over estimated and unstable in nature, as they resemble a brittle/unstable crack propagation. This can be observed in both the *P*-δ and the R-curves which completely distance themselves from the reference behaviour. All *P*-δ curves, after the crack starts to grow, present three stages, a first progressive crack propagation (Point 0 to 1), followed by a sudden drop (Point 1 to 2), and then another progressive crack growth stage (Point 2 to 3). On the R-curves, three stages are also present, an increase until the first peak (Point 0 to 1), then a sudden drop in energy (Point 1 to 2) and then another increase until the final plateau (Point 2 to 3). For each curve, these phenomena happen at different and later stages, evidencing a relation between them and ${a_{0}}_{\mathrm{ELS}}$.

**Figure 20:**
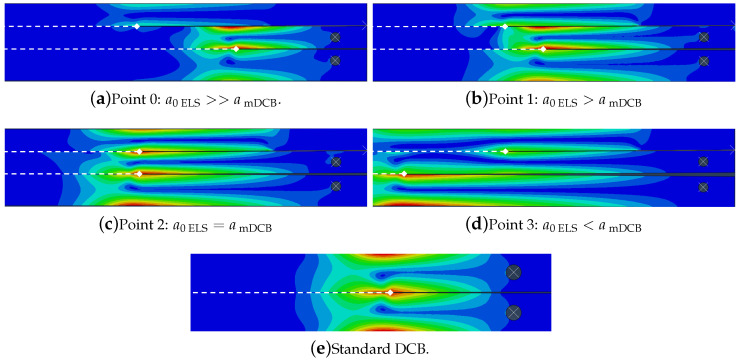
Stress distributions associated with the variation of ${a_{0}}_{\mathrm{ELS}}$ and ${a_{0}}_{\mathrm{mDCB}}$ configurations, plus the standard DCB stress distribution for reference. The dashed white line easily identifies each adhesive layer, as well as the evolution of the crack tips.

This becomes clearer when analysing the stress distributions associated with the complete crack growth of one of these specimen, defined by the actual crack length, $a_{\mathrm{mDCB}}$. This evolution is presented in Figure 20: starting at Figure 20a, where ${a_{0}}_{\mathrm{ELS}}$ is much larger than $a_{\;\mathrm{mDCB}}$; then going to Figure 20b, where ${a_{0}}_{\mathrm{ELS}}$ is larger than $a_{\;\mathrm{mDCB}}$, but is reaching the vicinity of the ELS crack; then at Figure 20c, where ${a_{0}}_{\mathrm{ELS}}$ is equal to $a_{\;\mathrm{mDCB}}$; and finally at Figure 20d, where ${a_{0}}_{\mathrm{ELS}}$ is smaller than $a_{\;\mathrm{mDCB}}$, which continues growing. Additionally, Figure 20e, is presented to show the stress distribution as a standard, continuous and symmetric specimen, since the mDCB is highly unsymmetrical and discontinuous.

Now, following the stages of crack growth previously presented, it is clearly seen that initially the ELS crack presents an unsymmetrical stress distribution, characteristic of this specimen, showing that the middle substrate is the most heavily loaded one. Initially, the crack growth evolution transitions from Point 0, the beginning of crack propagation, Figure 20a, until Point 1, where the crack reaches the vicinity of the ELS crack—Figure 20b.

While $a_{\;\mathrm{mDCB}}$ tends to equal ${a_{0}}_{\mathrm{ELS}}$, the stress concentrations associated with each crack tip start to interfere with each other, as can be clearly seen in Figure 20b (Point 1) to Figure 20c (Point 2). The transition between $a_{\;\mathrm{mDCB}}$ being smaller than ${a_{0}}_{\mathrm{ELS}}$ to $a_{\;\mathrm{mDCB}}$ being larger than ${a_{0}}_{\mathrm{ELS}}$ is then associated with the transition from Point 1 to 3, with 2 in the middle.

In the end, when the mDCB crack passes the stationary ELS crack, the last crack propagation propagation stage occurs for the *P*-δ curve, reaching the final $G_{IC}$ plateau for the R-curve.

##### Initial Crack Length Study—${a_{0}}_{\mathrm{ELS}}\leq {a_{0}}_{\mathrm{mDCB}}$

**Figure 21:**
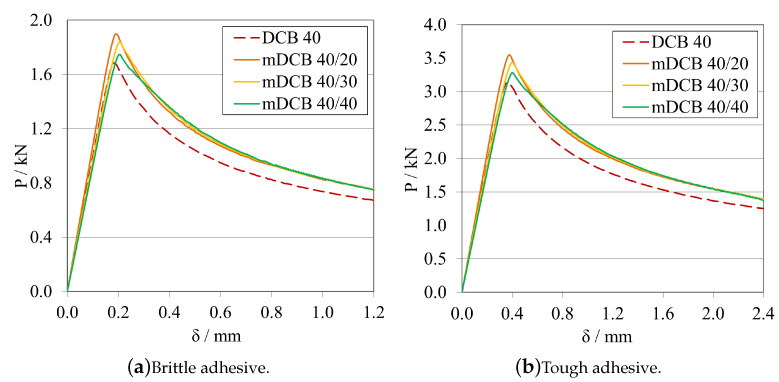
*P*-δ curves related to ${a_{0}}_{\mathrm{ELS}}\leq {a_{0}}_{\mathrm{mDCB}}$, compared against the correspondent standard DCB.

**Figure 22:**
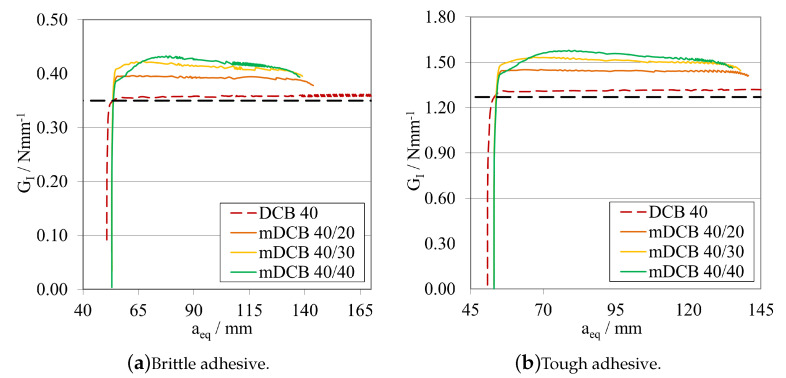
R-curves related to ${a_{0}}_{\mathrm{ELS}}\leq {a_{0}}_{\mathrm{mDCB}}$, compared against the correspondent standard DCB. Black dashed line represents the input $G_{IC}$ value.

Leaving the last subsection with the notion that having an ${a_{0}}_{\mathrm{ELS}}$ larger than ${a_{0}}_{\mathrm{mDCB}}$, was not recommended, the study naturally progressed towards an analysis of the opposite behaviour, ${a_{0}}_{\mathrm{ELS}}$ equal or even smaller than ${a_{0}}_{\mathrm{mDCB}}$. As such, a similar procedure was defined: ${a_{0}}_{\mathrm{mDCB}}$ was kept constant at 40 mm for both adhesives; and, for each adhesive, three ${a_{0}}_{\mathrm{ELS}}$ were tested, the values in question are present in the plot’s legend codes. Additionally, the same standard DCB was introduced in the plots to define a reference behaviour. The *P*-δ curves and R-curves, analysed by the standard DCB CBBM, are presented in Figure 21 and Figure 22a, for the brittle adhesive, and Figure 21 and Figure 22b, for the tough adhesive.

When analysing these configurations, the previously identified waviness phenomenon is not present. However, a similarity between the ${a_{0}}_{\mathrm{ELS}}={a_{0}}_{\mathrm{mDCB}}$ curves and the final stage of the tests with ${a_{0}}_{\mathrm{ELS}}>{a_{0}}_{\mathrm{mDCB}}$ can be seen, i.e., Points 2 to 3, where after crack growth initiation $G_{I}$ increases progressively until it reaches a plateau. This phenomenon starts to disappear as we pass from 10 mm of relative distance between crack tips, to 20 mm. This last case presented great similarities to the standard DCB test, showing good parallelism between them and a clear, long and stable plateau. Nevertheless, all $G_{IC}$ values where overestimated.

At this point, having overestimated the values of $G_{IC}$, a the novel mDCB CBBM method was developed (Appendix A) and employed for all further mDCB specimens, as an attempt to correct this behaviour.

This said, having removed the energy component absorbed by the ELS adhesive layer through this method, it was now also possible to see its evolution as the mode I crack propagates. As such, this variable ($G_{II}$) was employed for each tested condition in the following R-curve plots, represented as dashed double-dotted lines with the same codification as before, but now for the "ELS" test type.

**Figure 23:**
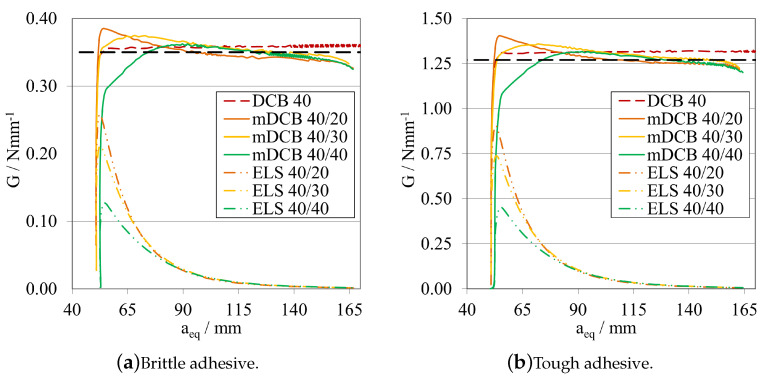
R-curves related to ${a_{0}}_{\mathrm{ELS}}\leq {a_{0}}_{\mathrm{mDCB}}$, compared against the correspondent standard DCB, analysed through the novel mDCB CBBM data reduction scheme. Black dashed line represents the input $G_{IC}$ value.

The R-curves, analysed by the novel mDCB CBBM, are presented in Figure 23a, for the brittle adhesive, and Figure 23b, for the tough adhesive.

By employing this novel method, all configurations presented a relatively stable plateau for the mid to end stage of crack propagation, at their actual respective $G_{IC}$. This proved that the method correctly estimated the energy release rate in pure mode I, by removing the energy absorbed by the ELS adhesive layer. However, all tests present certain particularities: for equal crack lengths, propagation starts prematurely for a smaller $G_{I}$ and then progresses towards the right value as $a_{eqI}$ gets farther away from $a_{0II}$, but never fully horizontally; for the mDCB 40/30, propagation starts at the right value but then increases slightly and starts to stabilise in the correct value right after, however it does not produce a fully horizontal line; and finally, for the 20 mm relative distance, propagation starts at an initial peak—possibly induced by the closeness of $a_{0II}$ to the loading point or the shortness of the ELS arm ($L^{*}=a_{eqI}$)—but quickly progresses towards the correct value with the most stable plateau, out of the three.

In terms of energy absorbed by the ELS adhesive layer, all configurations showed that $G_{II}$ increases rapidly until the start of crack propagation, where it begins to decrease since the arm of the ELS ($L^{*}$) increases in each instant, increasing $E_{fII}$, and as such reducing $G_{II}$.

As such, taking into consideration both the results from the completely stable plateaued but overestimated $G_{IC}$ of Figure 22, and the correct estimation of the energy release rate in mode I by using the mDCB CBBM formulation (Figure 23), a distance of 20 mm was set as the minimum value in the following tests.

##### Final Crack Length Relation—${a_{0}}_{\mathrm{ELS}}<{a_{0}}_{\mathrm{mDCB}}$

**Figure 24:**
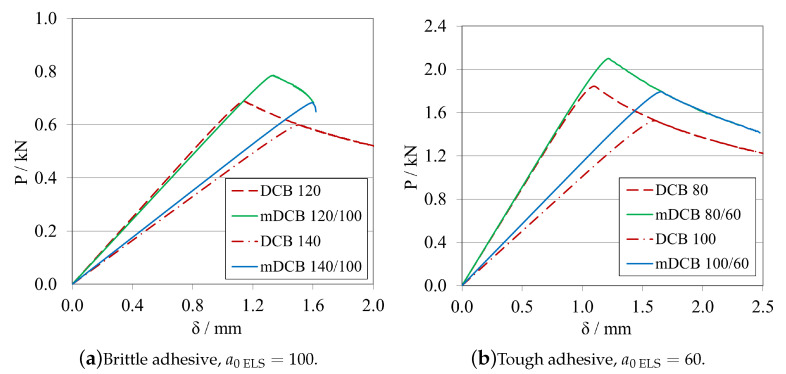
*P*-δ curves related to fixed ${a_{0}}_{\mathrm{ELS}}$, dependent on the adhesive, and ${a_{0}}_{\mathrm{ELS}}<{a_{0}}_{\mathrm{mDCB}}$, compared against the correspondent standard DCB.

**Figure 25:**
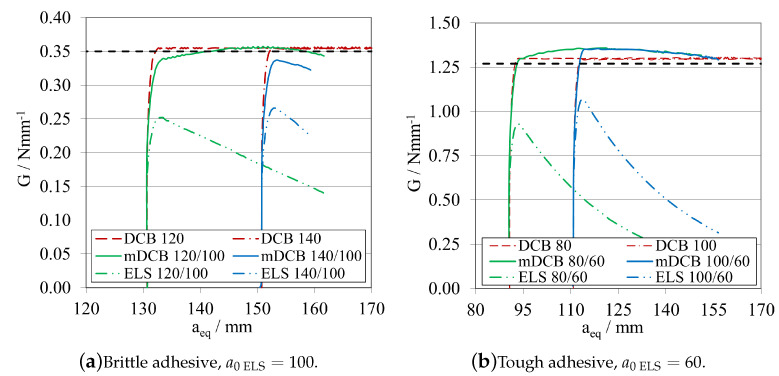
R-curves related to fixed ${a_{0}}_{\mathrm{ELS}}$, dependent on the adhesive, and ${a_{0}}_{\mathrm{ELS}}<{a_{0}}_{\mathrm{mDCB}}$, compared against the correspondent standard DCB, analysed through the novel mDCB CBBM data reduction scheme. Black dashed line represents the input $G_{IC}$ value.

Establishing that an ${a_{0}}_{\mathrm{ELS}}$ at least 20 mm smaller than ${a_{0}}_{\mathrm{mDCB}}$ is the best alternative, the following procedure was defined for ${a_{0}}_{\mathrm{ELS}}$ values more in account with the ones recommended in the ELS study. Now ${a_{0}}_{\mathrm{ELS}}$ was kept constant, at 100 mm for the brittle adhesive, and 60 mm for the tough adhesive; and, for each adhesive, two ${a_{0}}_{\mathrm{mDCB}}$ were tested to determine if larger distances between the crack tips would improve even further the performance of the specimen. The values in question are present in the plot’s legend codes. Additionally, two standard DCB with the same ${a_{0}}_{\mathrm{DCB}}$ used in the mDCB specimen were tested to define a reference behaviour for each mDCB condition. The *P*-δ curves and R-curves are presented in Figure 24 and Figure 25a, for the brittle adhesive, and Figure 24 and Figure 25b, for the tough adhesive.

By introducing the recommended values of ${a_{0}}_{\mathrm{ELS}}$, the tests showed better performances in relation to the previous study. This was mostly due to not having the initial peak on the R-curves that the mDCB 40/20 configurations showed before, as seen in Figure 23. Furthermore, the $G_{IC}$ values obtained were much more stable throughout the whole process, but with less propagation length, as expected. By increasing the relative difference between crack tips, the length of the plateau decreases, and in the case of the brittle adhesive mDCB 140/100 configuration it even reached the point where it does not have enough adhesive length to fully develop the FPZ and prematurely fails since it is too close to the end of the specimen.

Additionally, for the 20 mm of relative difference the stiffness of the mDCB test is much closer to the standard DCB, which is promising. As a result, it presented great similarities to the standard DCB test, showing good parallelism and a good stable plateau and only deviating from the standard test by its more rounded shape.

In the end, the combination of $\left[{a_{0}}_{\mathrm{ELS}}=100\;mm;{a_{0}}_{\mathrm{mDCB}}=120\;mm\right]$, is recommended for more brittle adhesives as it shows a good relation between a more stable ELS crack propagation and a long enough mDCB propagation length. For a more tough/ductile adhesive, the $\left[{a_{0}}_{\mathrm{ELS}}=60\;mm;{a_{0}}_{\mathrm{mDCB}}=80\;mm\right]$ is recommended to accommodate the larger FPZs.

##### Substrate Height Study—$h_{\mathrm{ELS}}$ and $h_{\mathrm{mDCB}}$

Establishing once more that ${a_{0}}_{\mathrm{ELS}}$ should be at least 20 mm smaller than ${a_{0}}_{\mathrm{mDCB}}$, the influence of the height of both substrates was tested. The following procedure was defined, where four combinations of $h_{\mathrm{ELS}}$ and $h_{\mathrm{mDCB}}$ were tested: the base configuration (Table 4) considered a balanced configuration since it presented a compliance ratio (*r*) of approximately 0.5, a second balanced configuration where both heights values were increased by 3.7 mm in relation to the base configuration; and two opposite unbalanced *h* relations, $h_{\mathrm{ELS}}$ equal to $h_{\mathrm{mDCB}}$ (12.7 mm), and $h_{\mathrm{mDCB}}$ equal to two times $h_{\mathrm{ELS}}$ (9 mm), which resulted in their *r* values being, respectively, 0.7 and 0.3.

**Figure 26:**
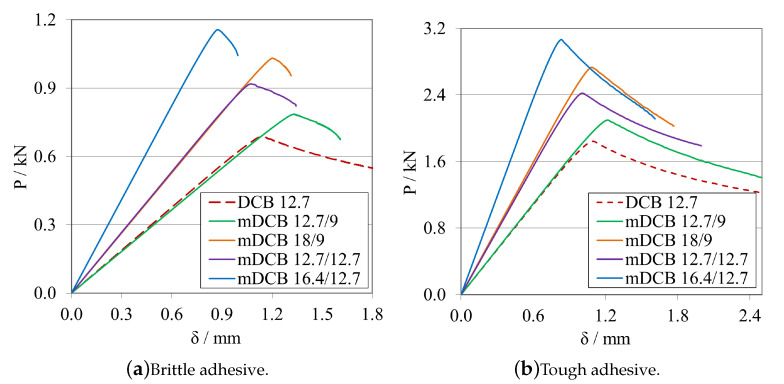
*P*-δ curves related to several combinations of $h_{\mathrm{ELS}}$ and $h_{\mathrm{mDCB}}$, compared against the correspondent standard DCB.

**Figure 27:**
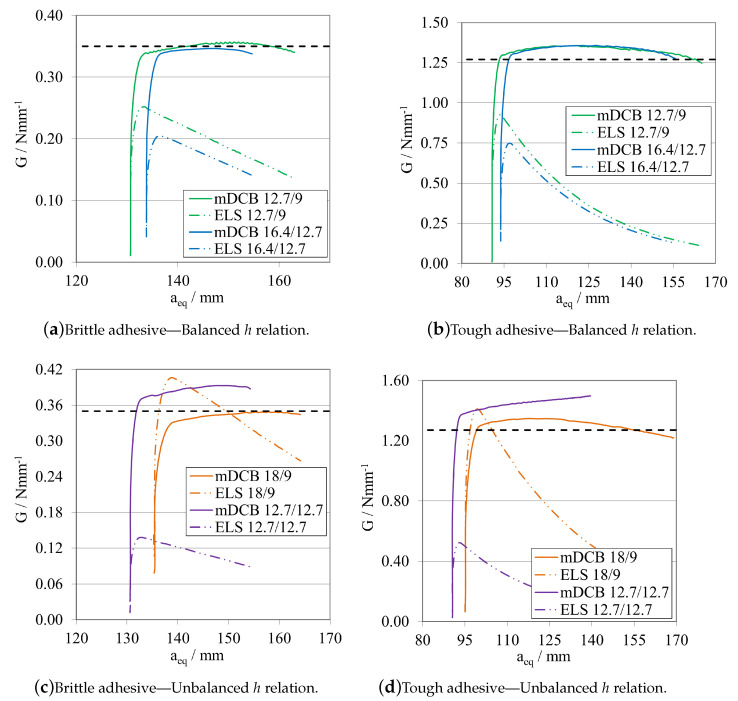
R-curves related to several combinations of $h_{\mathrm{ELS}}$ and $h_{\mathrm{mDCB}}$, compared against the correspondent standard DCB, analysed through the novel mDCB CBBM data reduction scheme. Black dashed line represents the input $G_{IC}$ value.

The *h* values in question were also presented in the plot’s captions. Additionally, a standard DCB with the same ${a_{0}}_{\mathrm{DCB}}$ was introduced in the plots to define a reference behaviour. The *P*-δ curves and R-curves are presented in Figure 26 and Figure 27a plus Figure 27c, for the brittle adhesive, and Figure 26 and Figure 27b plus Figure 27c, for the tough adhesive.

As a general conclusion of the height analysis, the base configuration was found to present the best results, with a more stable and longer plateau. However, some remarks can be made about the remainder of the results of this study. For the second balanced configuration, mDCB 16.4/12.7, the results were quite similar to the base configuration; however, this configuration results in a stiffer ELS specimen due to its higher $h_{ELS}$.

The unbalanced configurations presented different results, the mDCB 12.7/12.7 presented an initially stable propagation, slightly overestimated, but then the $G_{I}$ values increased progressively, similarly to what happened when equal crack lengths were used in the mDCB, and ELS specimens. For the mDCB 18/9 combination, the results were more similar to those of the balanced configurations in terms of $G_{I}$ evolution. However, higher values of $G_{II}$ were obtained, which, when changing to experimental tests should result in this effect being increased and possibly introducing a premature development of an FPZ in the ELS adhesive layer, hindering proper mode I adhesive characterisation.

##### Validation for Flexible/Ductile Adhesives

**Figure 28:**
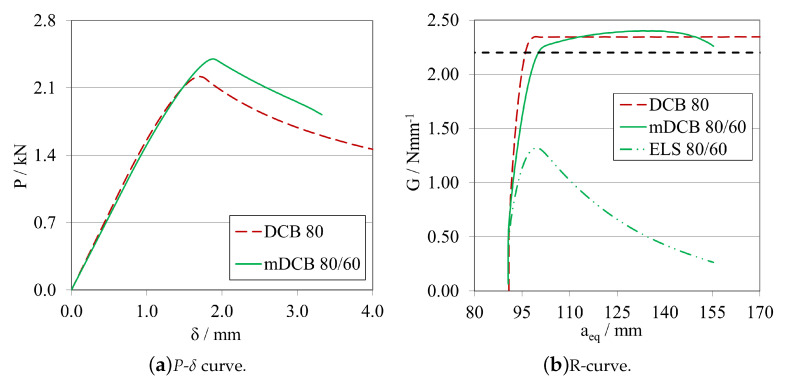
Curves related to the mDCB test of a generic flexible/tough structural adhesive, against the correspondent standard DCB, analysed through the novel mDCB CBBM data reduction scheme. Black dashed line in (**b**) represents the input $G_{IC}$ value.

A final analysis was conducted to ensure that the stiffness similarity between the two adhesives tested was not influencing the conclusions drawn throughout this work. For this purpose, a small study was carried out considering an adhesive three times less stiff and a two times more ductile adhesive than the previously considered tough adhesive. The *P*-δ curve and R-curve are presented in Figure 28a,b, respectively.

In the end, the same behaviour was found, where the *P*-δ curves present quite satisfactory parallelism, with a good similarity between the R-curves of the DCB reference and the mDCB test. The peak energy absorption in the ELS adhesive layer was around 50% of the $G_{IC}$, but far below the $G_{IIC}$ of this adhesive.

It should be noted that the more ductile the adhesive is, the more significant is the influence of the stress discontinuity present in the adhesive layer of the top bar of the mDCB specimen, i.e., the ELS specimen, especially when considering future experimental tests. This last test demonstrated that the final specimen dimensions proposed present a good characterisation performance for both mode I and mode II, encompassing a wide range of adhesives to be tested.

## 4. Conclusions

In this work, a study was conducted to better understand the parameters that govern the operation of a unified specimen for characterising adhesives under mode I (mDCB) and mode II (ELS) conditions. First, it was possible to develop a numerical model that precisely reflected the experimental conditions of the isolated end-loaded split test, and its geometry could be optimised by studying behavioural changes when computing different combinations of relevant geometry parameters. As a final summary, the following conclusions can be drawn for the ELS test:

- The mid span length, $d_{\mathrm{ELS}}$, is the most important parameter since it is related to a proper full development of the fracture process zone. This parameter should be large enough to prevent the FPZ reaching the vicinity of the clamping tool before its full development, as seen in Section 3.1.2. This can be tailored depending on the adhesive, higher values for more ductile adhesives, and vice versa for brittle adhesives.
- As a general rule, a sufficiently high $L_{\mathrm{ELS}}$—that is experimentally possible—should be used to allow a more stable crack propagation for a wide range of adhesives. As can be observed in Figure 10 and Figure 11, for lower values of $L_{\mathrm{ELS}}$, an unstable crack propagation occurs for both adhesives, and no plateau region can be achieved in the R-curves.
- By fixing $L_{\mathrm{ELS}}$, the useful adhesive length can then be changed by the initial crack length. As such, higher ${a_{0}}_{\mathrm{ELS}}$ values are recommended for brittle adhesives to improve the stability of the test, and vice versa for ductile adhesives to allow full development of the FPZ—as seen in Figure 8 and Figure 9.
- The height of the specimen interacts directly with both propagation stability (better for lower values) and substrate plasticity phenomenon (better for higher values), demonstrating opposite effects. The maximum load, for both adhesives, more than doubled when the height changed from 9 mm to 16.4 mm, while there was also a significant increase in the stiffness value. As such, a compromise between both phenomena should be chosen.

Afterwards, the same test procedure was applied to the modified double cantilever beam test, by applying the main conclusions of the mode II analysis, and then computing behavioural changes as a function of parameter changes. The most important conclusions of the mDCB test are then:

- The modified DCB test must have a relation of crack lengths where ${a_{0}}_{\mathrm{ELS}}$ is smaller than ${a_{0}}_{\mathrm{mDCB}}$, recommending a minimum relative distance of 20 mm, to prevent all the phenomena presented in Figure 18, Figure 19, Figure 20, Figure 21, Figure 22 and Figure 23. This is done to isolate the mDCB crack tip and reduce interference of the stress concentration associated with the ELS specimen’s crack tip on the mode I test—Figure 20.
- By using the novel CBBM formulation as the data reduction scheme of the modified DCB test, it was possible to obtain an R-curve with a considerably stable plateau that resulted in good estimations of the $G_{IC}$ values—Figure 23, Figure 25 and Figure 27.
- When considering the height of the substrates, a balanced configuration (r≈0.5) should be preferred, as the characterisation procedure presented better results for these configurations—Figure 27.

Overall, the base configuration with new initial crack tip relations for each adhesive type proved to be the best and final geometric configuration of this combined specimen—Table 6.

In the end, the present work allowed us to reach several conclusions that demonstrate the feasibility of this new specimen. Thus, the next step will be to experimentally validate the numerical predictions presented. Both the ELS specimen, and novel mDCB specimen plus the new CBBM formulation should be experimentally tested, by characterising a wide range of different adhesives in order to understand the limitations of this method. Furthermore, to do so may possibly even require the development of an NLFM approach such as a J-integral formulation, to better characterise highly ductile adhesives.

This will be a necessary and important step to move forward in the development of the final specimen, one where the four tests can be performed simultaneously obtaining both strength and fracture properties, defining a full cohesive law of an adhesive. Nevertheless, the unified mode I (mDCB) plus mode II (ELS) specimen has proved effective in characterising the two fracture adhesive properties in one single test.

## Figures and Tables

**Table 1:** Material properties used in the numerical study.

Property	Brittle Adhesive	Tough Adhesive ${}^{3}$	Steel
*E* / MPA	4890 ${}^{1}$	3880	210,000
ν / −	−	−	0.3
*G* / MPA	1560 ^1^	1447	−
$\sigma_{f}$ / MPA	41.0 ^1^	65.6	−
$\tau_{f}$ / MPA	30.2 ^1^	41.6	−
$G_{IC}$ / $Nmm^{-1}$	0.35 ^2^	1.27	−
$G_{IIC}$ / $Nmm^{-1}$	2.40 ^2^	7.22	−

Obtained: ${}^{1}$ [45]; ${}^{2}$ for this study, procedures from [46]; ${}^{3}$ [46].

**Table 2:** Boundary conditions used in the numerical study.

Boundary Conditions	Mode I Test	Mode II Test
$BC_{1}$	{0, 0, -}	Deactivated
$BC_{2}$	{0, $u_{y,I}$, -}	{0, $u_{y,II}$, -}
$BC_{3}$	{-, -, 0}	{-, 0, 0}
**Interactions**	**Mode I Test**	**Mode II Test**
PTFE	Surf. to surf. contact	Surf. to surf. contact

**Legend:**$u_{i}$ (Active) / 0 (Blocked) / - (Free)

**Table 3:** Base geometrical parameters of the ELS specimen. Dimensions in millimetres.

$L_{\mathrm{ELS}}$	${a_{0}}_{\mathrm{ELS}}$	$h_{\mathrm{ELS}}$	*b*	$L_{\mathrm{Clamp}}$	*t*
290	40	12.7	25	70	0.2

**Table 4:** Base geometrical parameters of the mDCB specimen. Dimensions in millimetres.

$L_{\mathrm{ELS}}$	$L_{\mathrm{mDCB}}$	${a_{0}}_{\mathrm{ELS}}$	${a_{0}}_{\mathrm{mDCB}}$	$h_{\mathrm{ELS}}$	$h_{\mathrm{mDCB}}$
290	190	40	40	9	12.7

**Table 5:** Base geometrical parameters of the DCB specimen. Dimensions in millimetres.

$L_{\mathrm{DCB}}$	${a_{0}}_{\mathrm{DCB}}$	$h_{\mathrm{DCB}}$
290	40	12.7

**Table 6:** Final recommendations for the geometrical parameters of the unified specimen. Dimensions in millimetres.

$L_{\mathrm{ELS}}$	$L_{\mathrm{mDCB}}$	${a_{0}}_{\mathrm{ELS}}$	${a_{0}}_{\mathrm{mDCB}}$	$h_{\mathrm{ELS}}$	$h_{\mathrm{mDCB}}$	$L_{\mathrm{Clamp}}$	*b*	*t*
290	190	100 / 60	120 / 80	9	12.7	70	25	0.2

**Figure A1:**
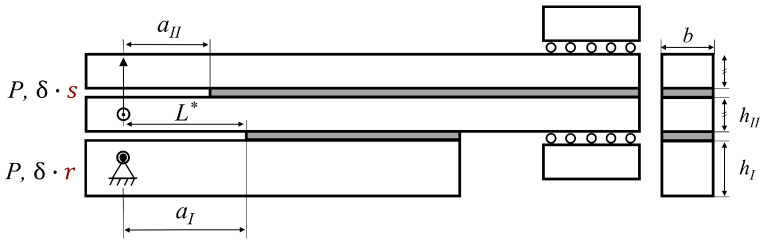
Schematic representation of the mDCB’s mechanical and dimensional parameters needed to deduce the compliance-based beam method (CBBM) data reduction scheme.

## Appendix A

Following the need to analyse the mDCB specimen through a custom data reduction scheme, the CBBM formulation was adapted for use with the modified DCB, taking into account the particularities of this specimen and the procedures used for the isolated DCB [6], ELS [11] and asymmetrical DCB [27] specimens. To simplify the equations in terms of variables, from now on, parameters associated with the mDCB adhesive layer or mDCB arm were addressed as “*I*” and similarly for the ELS they were referred to as “II”.

It is important to note that this method is solely valid for the cases where the crack lengths present the following relation: $a_{I}\geq a_{II}$. The mechanical and dimensional variables used for this procedure are shown in Figure A1.

In terms of variables, *P* and δ are the applied load and respective displacement, where *r* and *s* define the percentage of displacement supported by each part of the specimen, the lower beam (mDCB—*r*) and the upper composed beam (ELS—*s*).

Dimensionally, *b* is the specimen width, $h_{I}$ the thickness of the mDCB substrate, $h_{II}$ the thickness of the ELS substrates, $a_{I}$/$a_{II}$ the mode I and II crack lengths and finally $L^{*}$, which is defined as an auxiliary parameter that represents the ELS arm length until the mode I crack tip. Since, at any instance while $a_{I}$ is growing, the ELS arm length can only be defined up until the crack tip, $L^{*}$ is changing as the crack propagates, and so, it is equal to $a_{I}$.

Starting with the equation of strain energy (Equation (A1)) due to bending and shear effects:

(A1) $$U=2\left[\int_{0}^{L^{*}}\frac{M_{f}^{2}}{2EI}\;dx+\int_{0}^{L^{*}}\int_{-h/2}^{h/2}\frac{\tau^{2}}{2G_{13}}b\;dz\;dx\right]\;\wedge \;\tau =\frac{3}{2}\frac{V}{bh}\left(1-\frac{z^{2}}{c^{2}}\right)$$

where $M_{f}$ is the bending moment, *I* the second moment of area of each beam, *E* and $G_{13}$ the elastic properties of the substrate material. As for parameters *c* and *V*, these represent, respectively, the half-thickness and the transverse load on each beam ($0<x<L^{*}$).

Employing Equation (A1) in the problem at hand, and then applying the Castigliano theorem, results in Equation (A2), the total compliance of the specimen *C*. Being a summation of two components, the lower beam (DCB formulation), on the left, and upper composed beam (ELS formulation), on the right.

(A2) $$C=\left(\frac{4a_{I}^{3}}{E_{I}bh_{I}^{3}}+\frac{6a_{I}}{5G_{I}bh_{I}}\right)+\left(\frac{L^{*\;3}+3a_{II}^{3}}{2E_{II}bh_{II}^{3}}+\frac{3L^{*}}{5G_{II}bh_{II}}\right)$$

By this point, it is possible to substitute the auxiliary variable, $L^{*}$, with the actual crack length $a_{I}$, as this variable has served its purpose. Furthermore, $a_{II}$ can be substituted with its initial crack length value ($a_{0II}$).

Considering CBBM, the next step is to define the corrected flexural modulus, $E_{f}$, to take into account stress concentration and the substrates’ rotation near the crack tip [6]. However, for this combined specimen two moduli are present in the compliance equation, $E_{fI}$ and $E_{fII}$.

As such the total compliance was divided into two components, $C_{0I}$ and $C_{0II}$, considering that both the lower and upper beams sustain the same load (*P*) but absorb a part of the total displacement (δ) in relation to the crack tip. This ratio (Equation (A3)) is dependent of the asymmetry of the specimen which can be easily tracked, both numerically and experimentally, by the rotation (θ) of each loading point, in the initial linear-elastic stage, pre-propagation.

(A3) $$r=\frac{|\theta_{I}^{0}|}{|\theta_{II}^{0}\left|+\right|\theta_{I}^{0}|}\wedge s=1-r$$

This resulted in the following equations for the corrected flexural moduli in mode I (Equation (A4)):

(A4) $$E_{fI}={\left(C_{0I}-\frac{6\left(a_{0I}+\left|\Delta \right|\right)}{5G_{I}bh_{I}}\right)}^{-1}\frac{4{\left(a_{0I}+\left|\Delta \right|\right)}^{3}}{bh_{I}{}^{3}}$$

with Δ being the root rotation correction on the initial crack length ($a_{0I}$) [24].

Furthermore, similarly for mode II (Equation (A5)):

(A5) $$E_{fII}^{i}={\left(C_{0II}-\frac{3a_{eqI}^{i-1}}{5G_{II}bh_{II}}\right)}^{-1}\frac{{\left(a_{eqI}^{i-1}\right)}^{3}+3a_{0II}^{3}}{2bh_{II}^{3}}$$

where $a_{eqI}$ is the mode I equivalent crack length, that has not been defined at this point, so an iterative method was employed. As such, for each instant *i*, the value used was the equivalent crack length calculated in the previous instant (i−1). The start up value of $a_{eqI}$, for i=0, was the initial crack length ($a_{0I}$) plus the root rotation correction (Δ).

The next step was to define the equivalent crack length ($a_{eqI}$), by solving the following depressed cubic Equation (Equation (A6)), derived from Equation (A2):

(A6) $$\alpha a_{eqI}^{3}+\beta a_{eqI}+\gamma =0$$

which can be solved by the Cardano formula.

Considering that both the mDCB and ELS adhesive layers are absorbing energy during this test, and using the Irwin–Kies equation, the fracture energy obtained concerns the total energy absorbed by the specimen (Equation (A7)), and as such by itself does not represent the energy release rate of the adhesive in mode I loading conditions.

(A7) $$G_{T}=\frac{P^{2}}{2b}\left(\left(\frac{12a_{eqI}^{2}}{E_{fI}bh_{I}^{3}}+\frac{6}{5G_{I}bh_{I}}\right)+\left(\frac{3a_{eqI}^{2}+9a_{0II}^{2}}{2E_{fII}bh_{II}^{3}}+\frac{3}{5G_{II}bh_{II}}\right)\right)$$

However, considering Equation (A2), it is possible to identify a component dependent on the initial crack length of the ELS specimen, $a_{0II}$. Therefore, by removing this component from the total energy, $G_{T}$, it is possible to isolate the energy absorbed to propagate the mDCB crack, $G_{I}$ (Equation (A8)):

(A8) $$G_{I}=G_{T}-G_{II}\;\wedge \;G_{II}=\frac{9a_{0II}^{2}P^{2}}{4E_{fII}b^{2}h_{II}{}^{3}}$$

This method continues to not require monitoring of the crack length during propagation, being calculated as a function of the specimen compliance during the test, considering also the FPZ effects on the measured fracture energy.
